# Calcium coordination polymer containing dimethylphosphate ligands and exhibiting nucleating properties towards α and Β crystal polymorphs of isotactic polypropylene

**DOI:** 10.1038/s41598-025-99757-4

**Published:** 2025-05-02

**Authors:** Maciej Dębowski, Mateusz Kullas, Krystyna Czaja, Beata Sacher-Majewska, Marcin Bączek, Maciej Dranka, Andrzej Ostrowski, Zbigniew Florjańczyk

**Affiliations:** 1 Faculty of Chemistry, Warsaw University of Technology, Noakowskiego 3, Warsaw, 00-664 Poland; 2 Institute of Chemistry, University of Opole, Oleska 48, Opole, 45-052 Poland; 3 Faculty of Materials, Civil and Environmental Engineering, University of Bielsko-Biała, Willowa 2, Bielsko-Biała, 43-309 Poland

**Keywords:** Hybrid polymer, Calcium diorganophosphate, Isotactic polypropylene, Crystallization, Nucleating agent, Polymer composite, Coordination chemistry, Materials chemistry, Coordination polymers, Mechanical properties, Polymer characterization, Solid-state chemistry

## Abstract

The synthesis, structure and thermal properties of a one-dimensional coordination polymer based on calcium bis(dimethylphosphate) (CaDMP) are reported. The rod-like particles of CaDMP crystallized from water in a monoclinic space group *P*2_1_/*c*, and contained Ca[O_2_P(OCH_3_)_2_]_2_ polymeric chains consisting of the octahedrally coordinated Ca^2+^ ions bridged by the tridentate dimethylphosphate ligands. The variable temperature powder X-ray diffraction measurements showed that this structure undergoes anisotropic thermal expansion upon heating, with no polymorphic transitions occurring up to 190 °C. Thermolysis of CaDMP began around 260 °C leading to the formation of calcium condensed phosphates and volatile oxophosphorus species. A detailed differential scanning calorimetry (DSC) analysis, combined with a fitting of the experimental data to the Avrami or Liu–Mo kinetic models, revealed that CaDMP accelerates isothermal and non-isothermal crystallization of isotactic polypropylene (iPP). DSC and wide-angle X-ray scattering measurements confirmed the presence of α-iPP and β-iPP crystal domains in the systems loaded with CaDMP particles. The crystallographic analysis indicated that β-iPP polymorph formed via epitaxial crystallization on the surface of CaDMP crystals. Mechanical tests proved that the CaDMP-containing composites exhibited better ductility and impact strength than neat iPP.

**Supplementary Information:**

The online version contains supplementary material available at 10.1038/s41598-025-99757-4.

## Introduction

Nowadays, nucleating agents (NAs) are very important components of many polymeric materials. They increase the crystallization rate of polymers and promote the formation of large amounts of small crystallites, which have a beneficial effect on the optical and mechanical properties of the polymer compositions. Moreover, a properly selected NA can also influence the structure of the resulting crystalline phase formed by the polymer matrix^1^. An example of such polymer is isotactic polypropylene (iPP), that can form several polymorphic crystalline phases (i.e., the α, β, γ, ε polymorphs) composed of identical 3_1_ helices exhibiting (TG)_3_ or (TG٭)_3_ conformation and periodicity of ca. 6.50 Å (3 iPP monomeric units within a single turn), but differing in a spatial packing of iPP macromolecules (i.e., packing polymorphism)^2^.

Under typical processing conditions used in industrial applications of iPP, a monoclinic polymorph of iPP (α-iPP) (cell parameters *a* = 6.65 Å, *b* = 20.96 Å, *c* = 6.50 Å, β = 99.3°; melting temperature of ca. 170 °C) is usually produced^2^. The α-iPP NAs include inorganic fillers (e.g., CaCO_3_, talc, wollastonite, or metal-doped silicates)^3–5^ or metal salts of cyclic carboxylic acids (e.g., benzoates^6^ or derivatives of hexahydrophthalic acid^7^. Other NAs that are able to significantly increase the crystallization temperature during melt crystallization of α-iPP and reduce its haziness at low concentrations are based on several groups of organic or metal-organic compounds such as acetalized cyclic sorbitol derivatives^8^, substituted aromatic heterocyclic organophosphates^4,8–10^, metal dicarboxylates^11,12^ and phosphonates^13,14^, metal complexes with glycerol^15^, substituted mono- and dihydrazides^16^, as well as some homologues of *N*,*N*′-dicyclohexylcarboxamide^17^ or trisamides^18^.

A metastable β form of iPP (β-iPP) is characterized by polymer helices packed within a trigonal unit cell described by the lattice parameters *a* = *b* = 11.03 Å and *c* = 6.50 Å, and has a melting point about 20 °C lower than α-iPP^2^. Although the appearance of β-crystals in iPP can be stimulated by crystallization under appropriately selected shear–stress conditions or temperature gradients, in order to obtain high contents of this phase, it is necessary to use special β-iPP nucleating agents (β-NAs)^1^. The most frequently applied are commercially available additives known under the trade names such as Hostaperm^®^ (γ-quinacridone)^19^, NJSTAR^®^ NU-100 and TMB-5 (*N*,*N*’-dicyclohexyl-substituted aryl bisamides)^20^, as well as rare earth metals-based WBG-II (a bimetallic complex of calcium and lanthanum having a general formula of Ca_x_La_1−x_(L1)_m_(L2)_n_, where L1 and L2 are dicarboxylic acids and amide-type ligands, respectively) and NAB-83 (a tetrahydrophthalic anhydride complex of selected divalent metals with an undisclosed composition)^21^. Various other additives having a significant β-iPP nucleating efficiency can be divided into several groups of chemical compounds: bisamides (e.g., secondary amides derived from aliphatic dicarboxylic acids^17,22^ and *N*-substituted derivatives of phthalamide bearing cyclohexyl or phenyl groups^22–25^, trisamides^26^, as well as metal salts of monocarboxylic acids (e.g., *p*-*n*-alkylbenzoate-alumoxanes^27^, acyclic aliphatic dicarboxylic acids (e.g., salts of divalent metals with malonic, glutaric, adipic, pimelic, suberic or azelaic acids)^12,28–31^, cyclic dicarboxylic acids^32,33^, or phthalic acid^34^ and its hydrogenated derivatives^7,35,36^. In addition, several inorganic compounds (e.g., surface-functionalized or neat metal oxides, hydroxides^37–41^ and salts^42,43^, neat or organically-modified silicate-type minerals (e.g., montmorillonite^44^, wollastonite^45^, and halloysite nanotubes^46^, metal particles (e.g., silver nanoparticles^47^ have also been reported as being capable of inducing the β-iPP crystallization under specific processing conditions. Interestingly, carbonaceous (nano)structures (i.e., pimelate-modified graphene nanoplatelets or graphene oxide particles^48,49^ and several organic polymers (e.g., liquid crystalline polymers^50^, hyperbranched polyesters^51^, biomimetic polydopamine^52^, or polystyrene^53^ exhibit similar nucleating properties towards β-iPP. A more detailed account of known β-iPP NAs can be found in a recent review by Wu et al.^54^.

β-iPP provides some advantageous thermal and mechanical properties of final materials, e.g. a higher heat distortion temperature, impact strength and toughness^1,55^, or better ductility and drawability^56^, in comparison to the products containing only the α-iPP domains. β-iPP also exhibits better resistance to both thermal and UV-induced photooxidative degradation^55^. However, its presence in iPP also has some drawbacks, the most important of which occurs under tensile stress conditions (i.e., a reduced values of Young’s modulus and yield stress) and can effectively limit industrial applications of the materials incorporating β-iPP phase^56–58^. An attractive option to solve these problems is to combine a stiffness of α-iPP with a toughness of β-iPP in one material, either thru utilization of a mixture of α-NA and β-NA (the so-called α/β compound NA)^59,60^, or a single NA exhibiting a dual nucleating ability (i.e., NA promoting the formation of both iPP polymorphs)^57,61,62^. It seems that the latter method is easier to apply in the industry since in most cases one can tailor the properties of the resulting material by proper changes of such processing conditions as the NA concentration in the iPP matrix and time-temperature protocols^57,61^, whereas in the case of the α/β compound NA one has also to control the α-NA/β-NA mass ratio and homogeneity of their mixture^60^. In fact, many of the up-to-date commercialized β-NAs exhibit dual nucleating activity^21^.

Several calcium salts of phosphoric acid diesters (CaDOPs) have been already described in the literature, for example those bearing aliphatic groups^63,64^, phenyl moieties^65,66^ or substituted aromatic rings^65,67–70^. However, a full crystallographic analysis has been carried out only for a few of them, namely those with [(^*t*^BuO)_2_PO_2_^−^]^64^, (PhO)_2_PO_2_^−^ or (*p*-NO_2_PhO)_2_PO_2_^−^ ligands^65^. It should be noted that in the case of homometallic systems (i.e., CaDOPs containing only calcium as the metallic centers of coordination), a common structural feature is the formation of 1D polymeric chains made of the octahedrally coordinated Ca^2+^ cations bridged by bidentate diorganophosphate groups and saturating their coordination sphere with oxygen ligands from water molecules^64,65.^ The potential applications of CaDOPs range from additives impeding biomineralization^65^, through components of ion-selective electrodes^67,68^ and catalysts in asymmetric organic synthesis^69^, to precursors of calcium phosphate-based ceramic materials^64^. In addition to that, their usefulness in polymer technology have also been verified, for example as catalysts in ring-opening polymerization of cyclic carbonates and lactones^66^, or the already mentioned α-iPP NAs^70^. Recently, we have found that calcium bis(dimethylphosphate) (CaDMP) is an effective additive for iPP that produces a significant amount of the β-iPP phase under non-isothermal conditions^71^, and acts as a flame retardant^72^.

In this paper, we report on the synthesis, crystallographic structure and thermal transitions of CaDMP, as well as present the results indicating its effect on the crystallization of iPP. To the best of our knowledge, CaDMP is the first structurally characterized CaDOP hybrid polymer that does not contain any auxiliary ligands, and can be successfully applied as the α/β nucleating agent for iPP. Studies on its structure provided data helpful in determining the role of epitaxy in the β-iPP nucleation process and helped establish the structural features at the root of the β-iPP polymorph formation.

## Experimental section

### Materials and reagents

Unless stated otherwise, all chemicals and reagents were purchased from commercial sources and used without further purification: trimethyl phosphate (TMP) (≥ 98%, Merck Schuchardt OHG), calcium carbonate (99+%, Sigma-Aldrich), diethyl ether (pure p.a., Chempur). iPP Moplen HF501N (a homopolymer without any additives, Melt Flow Index 10 g (10 min)^−1^, density 900 kg m) was provided by LyondellBasell and used as a polymer matrix to prepare composites.

### Preparative procedures

#### Synthesis of calcium dimethylphosphate

CaDMP was synthesized according to the following procedure: CaCO_3_ (30.00 g, 297 mmol), TMP (87.12 g, 609 mmol), and redistilled water (550 mL) were placed in a 1 L round-bottom flask equipped with a magnetic stirring bar and reflux condenser. The mixture was vigorously stirred and heated under reflux for 24 h. After the reaction completion and cooling to room temperature, the post-reaction mixture was filtered off and the clear and colourless filtrate was concentrated on a rotary evaporator at 60 °C, resulting in the formation of a large amount of white crystalline solid. After dispersing in Et_2_O (150 mL), the crude insoluble product was isolated via filtration on a Büchner funnel and washed with Et_2_O (3 × 50 mL). After a two-step drying procedure (24 h at 60 °C in a vacuum oven, followed by 6 h at 120 °C in an air dryer) 72.64 g of anhydrous CaDMP was obtained (reaction yield of 84%).

#### Preparation of neat iPP and its composites with CaDMP

Polypropylene materials filled with CaDMP were prepared by a two-step melt blending method. In the first step, the iPP masterbatches containing 20 wt% of CaDMP were prepared using a HAAKE PolyLab Rheometer (Thermo Fisher Scientific Inc., San Jose, CA, USA) working at 190 °C (rotor speed 100 rpm, duration 10 min.). In the second step, the obtained masterbatches were blended with a neat iPP powder at the predetermined mass ratios using a IM-15 laboratory conical twin screw extruder (ZAMAK, Skawina, Poland) (temperatures at 4 working zones: 170, 175, 180 and 185 °C, motor speed 100–200 rpm) coupled with a IMM-15 laboratory injection moulding machine (ZAMAK, Skawina, Poland) (barrel temperature 180 °C, injection pressure 7 MPa, form temperature 80 °C). The obtained iPP-based composites contained 0.2, 0.5, 1.0, 2.0 or 5.0 wt% of CaDMP. Henceforth, they will be denoted as iPP/xCaDMP, where x stands for the number describing content of CaDMP (in wt%) within the respective composite).

The sample of neat iPP was prepared from iPP powder by applying the same processing conditions as in the case of the composites.

### Characterization methods

#### Elemental analysis

The hydrogen and carbon contents were determined using a Vario EL III elemental analyzer (Elementar Analysensysteme GmbH, Langenselbold, Germany). The measurements were carried out at 1,150 °C under helium/oxygen atmosphere. The obtained results are presented in Table S1 in the Supplementary Information (SI).

#### Flame atomic absorption spectroscopy (FAAS)

The calcium content was determined by means of FAAS using Aanalyst 800 atomic absorption spectrometer (Perkin-Elmer, Inc., Shelton, Connecticut, USA). The atomization of the sample was carried out in the acetylene–nitrous oxide flame. Prior to the FAAS analysis the sample was subjected to a microwave mineralisation in nitric acid using a UltraWAVE apparatus (Milestone Srl, Sorisole, BG, Italy). The obtained results are presented in Table S1 in SI.

#### Single-crystal X-ray crystallography

For the purpose of the X-ray crystallographic analysis the clear, aqueous filtrate obtained from the post-reaction mixture was subjected to a slow, partial evaporation at room temperature in a stream of air. A single crystal suitable for the X-ray diffraction studies was selected from the thus crystallized solid under a polarizing microscope. Subsequently, it was mounted in inert oil and transferred to the cold gas stream of the κ-CCD Gemini A Ultra diffractometer (Oxford Diffraction Company, Wroclaw, Poland). Cell refinement, as well as data collection, reduction and analysis were performed with the CrysAlis^PRO^ software. The crystal data were processed in Olex2^73^, solved with the ShelXT structure solution program using Intrinsic Phasing^74^ and refined with the SHELXL−2018/3 program refinement package using least squares minimization^75^. The structure was refined using the hklf 5 routine on-merohedral twins resulting in a BASF value of 0.5044(1). The twin law is a twofold rotation axis around the [001] crystallographic direction in direct space. Hydrogen atoms were added to the structure model at geometrically idealized coordinates and refined as riding atoms. The experimental parameters and crystal data for CaDMP are summarized in Table S2, S3 and S4 in SI.

#### Powder X-ray diffraction (PXRD) analysis

PXRD pattern of CaDMP thermolyzate was recorded at room temperature on a D8 Advance automated diffractometer (Bruker, Karlsruhe, Germany) equipped with a Lynx-Eye position-sensitive detector using Cu-Kα radiation (*λ* = 1.5406 Å). The data were collected in the Bragg–Brentano (*θ*/2*θ*) horizontal geometry (flat reflection mode) between the 2*θ* angles of 8° and 60° in steps of 0.03°, with 2 s per step (total time 384 s).

#### Wide-angle X-ray scattering (WAXS) analysis

WAXS measurements were performed on a URD-6 diffractometer (Seifert-FPM GmbH, Freiberg, Germany) equipped with copper target X-ray tube (*λ* = 1.5418 Å) operated at 40 kV and 30 mA. Cu-Kα radiation was monochromized with a graphite filter. WAXS curves were recorded in a symmetrical reflection step scanning mode, over the 2*θ* range of 3–60°, with the step size of 0.1° and the registration time of 20 s per step, using a scintillation counter. The samples of iPP or its composites with CaDMP subjected to the WAXS measurements had the shape of the 100 μm-thick foils produced by pressing the previously injection-moulded items for 2 min between two copper plates (at 190 °C and 5 MPa) using a hand press QC677A (Cometech Testing Machines Co., LTD, Taichung, Taiwan), and subsequent cooling to room temperature for 5 min under the mass of 2 kg.

#### Variable temperature PXRD (VT-PXRD) analysis

VT-PXRD measurements above room temperature were carried out on a D8 Discover instrument (Bruker, Germany) equipped with a VANTEC-1 position-sensitive detector and a DCS-350 heating stage (Anton Paar) with a temperature stability of 1 K, using Cu-Kα radiation and a step size of 0.0122°. The data were collected between the 2*θ* angles of 3° and 60°.

#### Fourier-transform infrared (FTIR) spectroscopy

FTIR analysis was conducted on a Nicolet iS5 spectrometer equipped with an iD7 diamond attenuated total reflectance accessory (Thermo Electron Scientific Instruments LLC, Madison, WI, USA)). The sample or background spectra consisted of 16 scans (1.6 s per scan).

#### Nuclear magnetic resonance (NMR) spectroscopy

^1^H and ^31^P NMR spectra were recorded in CD_3_OD on a Mercury 400 MHz spectrometer (Varian Inc., Palo Alto, CA, USA)) operating at 400.11 and 161.96 MHz, respectively. Chemical shifts are reported relative to the residual solvent signal (^1^H NMR) or 85% H_3_PO_4(aq)_ (^31^P NMR). The measurements were carried out at room temperature.

#### Solid-state magic angle spinning (MAS) NMR spectroscopy

Standard solid-state ^31^P cross-polarized MAS NMR measurements were conducted on an Avance III 400 Wide Bore spectrometer (Bruker, Germany) at 22 °C with a spinning rate of 8 kHz and resonance frequency of 161.98 MHz. Data processing was performed using Bruker TopSpin software.

#### Differential scanning calorimetry (DSC)

DSC measurements of CaDMP were carried out on a DSC Q200 apparatus (TA Instruments, New Castle, DE, USA). The following temperature program was applied: heating run from −150 °C to 220 °C followed by a cooling run to −150 °C and another heating run from −150 °C to 220 °C. During all heating/cooling runs the temperature was changed at a steady rate of 10 °C min^–1^.

DSC measurements of the injection-moulded samples of neat iPP or its composites with CaDMP were carried out on a Mettler-Toledo DSC1 differential scanning calorimeter (Mettler Toledo GmbH, Greifensee, Switzerland) under a nitrogen atmosphere. The sample weights were between: 2.89 and 5.19 mg (for non-isothermal measurements), 3.66 and 4.28 mg (for isothermal measurements), or 2.20 and 5.35 mg (for isothermal stepwise crystallization measurements). One of the following time–temperature programs was applied, depending on the type of the investigated crystallization process:

- for a non-isothermal crystallization: heating run from room temperature to 190 °C followed by a cooling run to 0 °C and another heating run from 0 °C to 190 °C. During all heating runs, the temperature was changed at a steady rate of 10 °C min^–1^, whereas the cooling run was carried out at a cooling rate prechosen from the 3–10 °C min^–1^ range;
- for an isothermal crystallization: heating run from room temperature to 200 °C at 10 °C min, isothermal run at 200 °C for 15 min, followed by a cooling run to 132 °C with a rate of 20 °C min^–1^ and isothermal crystallization at that temperature for 939–5400 s (depending on the time required for a complete evolution of the crystallization exotherm). After crystallization completion, the sample was heated to 200 °C at the 5 °C min^–1^ rate. Please note that during a cooling run no first- or second-order thermal transitions were observed even for the composite system with the highest CaDMP content;
- for a stepwise isothermal crystallization: heating run from room temperature to 200 °C at 10 °C min^–1^, isothermal run at 200 °C for 15 min, followed by a cooling run to 132 °C with a rate of 20 °C min^–1^ and isothermal crystallization at that temperature for a predefined time. Subsequently, the temperature was increased to 140 °C at a rate of 20 °C min^–1^ and then to 200 °C at a rate of 5 °C min^–1^.

#### Thermogravimetry (TG) in air

TG analysis of CaDMP was performed in a stream of synthetic air using a Mettler-Toledo TGA/DSC1 thermogravimetric analyser equipped with a SDTA sensor (Mettler Toledo GMBH, Greifensee, Switzerland). The sample (12.018 mg) was heated from 30 °C to 900 °C, at a heating rate of 10 °C min^–1^.

#### Simultaneous thermal analysis (STA)

Volatile thermal decomposition products and thermal effects of the processes occurring during heating were studied by TG coupled with differential thermal analysis (DTA) and quadrupole mass spectrometry (QMS) using a STA 449 C Jupiter apparatus (Netzsch, Selb, Germany) coupled with a QMS 403 C Aeolos quadrupole mass spectrometer (Netzsch, Selb, Germany). The sample was placed in Al_2_O_3_ crucible and heated in a stream of argon (flow rate of 90 mL min^–1^) from 30 °C to 1,000 °C at a heating rate of 5 °C min^–1^.

#### Scanning electron microscopy (SEM)

SEM images of the samples were taken using a Helios 5 PFIB scanning electron microscope (Thermo Fisher Scientific, Hillsboro, OR, USA) equipped with a high-resolution Elstar field emission electron column, a Phoenix ion column with inductively coupled Xe^+^ plasma, and a set of Elstar detectors for secondary electrons (standard ETD detector placed in the chamber) and intra-column TLD (for high-resolution immersion imaging), ABS/CBS scatter electrons detector, STEM3 + detector, and ICE detector for ion imaging. During the measurements, the working distance of ca. 4.0 mm and the accelerating voltage of the electron beam of 5 kV were used.

Before the SEM measurement, all samples were sputtered with a 10 nm-thick Au layer using a CCU-010 high-vacuum sputtering machine (Safematic GmbH, Zizers, Switzerland).

#### Mechanical tests of the injection-moulded specimens

Tensile tests based on the PN-EN ISO 527 standard were carried out on an Instron 33R universal machine (Instron, Norwood, MA, USA), with a crosshead speed of 100 mm min^–1^. The results presented in the manuscript are the average of the measurements of 3 samples.

Charpy impact tests based on the PN-EN ISO 179 standard were conducted on a Zwick HIT 50P apparatus (Zwick Roell, Ulm, Germany) equipped with the 0.5 J pendulum. The results presented in the manuscript are the average of the measurements of 3 samples.

## Results and discussion

### CaDMP synthesis and thermal stability

CaDMP was synthesized according to a modified method that previously had been utilized successfully by our research group in the case of its zinc-containing analogue^76^. Compared to the methods already reported in the literature, our hydrothermal pathway offered significant advantages since it proceeded with a very high yield (typically exceeding 80%), utilized commercially available, non-hazardous reagents, and did not require any special reaction conditions or apparatus (e.g., inert atmosphere, anhydrous organic solvents, pressure vessels). It was based on a simple acid-base type reaction taking place in aqueous medium between CaCO_3_ and dimethyl phosphate generated in situ via hydrolysis of one phosphoester group from TMP molecule. The intermediate product, namely CaDMP hydrate, easily underwent dehydration to anhydrous CaDMP even at room temperature, but drying in a stream of hot air helped to complete that process.

The presence of only one type of ligands (e.g., (CH_3_O)_2_PO_2_^−^ groups) within CaDMP structure was confirmed by the results of elemental analysis (carbon, hydrogen and calcium contents, see Table S1 in SI), which showed a very good agreement (e.g., up to 4.4% of a relative difference) with the values theoretically calculated for the polymer with a general formula of Ca[O_2_P(OCH_3_)_2_]_2_. FTIR spectrum of CaDMP (Fig. S1 in SI) also lacked any absorption bands related to a free or coordinated water, such as those ascribed to either the O–H stretching (a spectrum region above 3,000 cm^–1^) or HOH bending (at ca. 1,640 cm^–1^) modes. It corresponded very well to the infrared absorption spectrum of barium dimethylphosphate^77^, showing the presence of the aliphatic C–H stretching and bending bands (2,840–3,000 cm^–1^ and 1,440−1,465 cm^–1^ regions, respectively), as well as four absorption signals located at 1,230, 1,088, 806 and 743 cm^–1^ that could be ascribed to the asymmetric and symmetric stretching vibrations within the phosphate moiety: the first two of them arise from the P–O bonds involved in calcium coordination, whereas those below 1,000 cm^−1^ were related to the phosphoester linkages (their spectral position is less sensitive to the change of the metal cation in the analysed compound)^76–78^. The two most prominent bands observed on the FTIR spectrum of CaDMP (at 1,037 and 1,060 cm) resulted from the stretching modes of the C–O bonds^77,78^.

Further evidence of the purity of CaDMP was given by the observation of single resonance peaks on its ^31^P NMR spectra recorded either in the solid state (*δ*_P_ = 0.01 ppm, Fig. S2 in SI) or in solution (*δ*_P_ = 4.09 ppm, Fig. S3 in SI). These findings suggested that at the resolution of both ^31^P NMR methods all phosphorus nuclei of CaDMP are in the same chemical environment and no traces of an unreacted TMP, or products of further hydrolysis of phosphoester bonds could be detected. Interestingly, ^1^H NMR spectrum of CaDMP (Fig. S4 in SI) contained two peaks located at 3.58 and 3.55 ppm, with integrals of an equal value. We have previously reported the same phenomenon (e.g., “double” methyl protons’ NMR signal and one population of magnetically distinct phosphorus nuclei) in the case of zinc bis(dimethylphosphate) NMR spectra^76^, so it can be assumed that in a strongly polar solvent CaDMP exhibited a similar behaviour as the former: it underwent depolymerization to monomeric species or free ions. From that point of view, the occurrence of two ^1^H NMR methyl signals could result from phosphorus-proton spin-spin coupling, especially that such type of coupling via 3 bonds and characterized by the coupling constants around 10 Hz has been already reported for various dimethylphosphates, including barium salt^79^.

The results of TG analysis (Fig. S5 and Table S5 in SI) showed that in air CaDMP was thermally stable up to 260–294 °C, depending on the selected criterion of the beginning of its thermolysis: the lowest value characterized the temperature of a bending point on the TG curve (*T*_b_ = 257.7 °C), whereas both the extrapolated onset temperature (*T*_onset_ = 275.7 °C) and the temperature at which CaDMP sample lost the first 2% of its initial mass (*T*_98%_ = 279.4 °C) were higher. Oxidative degradation proceeded via several overlapping steps (a maximum rate of decomposition was observed around *T* = 299 °C) during which the sample lost almost half of its mass and transformed into a mixture of inorganic calcium condensed phosphates, among which the crystalline phases of calcium metaphosphate and calcium pyrophosphate could be identified by means of PXRD studies (see Fig. S6 in SI) – phase identification was performed using the PDF-2 database^80^. Interestingly, the quadrupole mass spectrometry (QMS) spectra of the gaseous products evolved during CaDMP pyrolysis (Fig. S7 in SI) showed a group of signals characteristic of the oxophosphorus species. A similar situation has been reported in the case of aluminum tris(dimethylphosphate) (AlDMP) pyrolysis^81^. In fact, the QMS spectra of CaDMP and AlDMP closely match each other suggesting that both these compounds undergo pyrolytic degradation according to the same set of chemical transformations, e.g., a homolytic P–OCH_3_ bond scission and subsequent rearrangements of the newly liberated methyl and methoxyl radicals, resulting in the formation of identical volatiles that included dimethyl ether, methanol, as well as TMP and its products of thermal degradation^81^. It is worth noting, that these findings have also been confirmed by the FTIR detection of TMP in the liquid phase obtained after a condensation of volatiles formed during CaDMP pyrolysis in nitrogen^72^.

### Crystal structure of CaDMP and unit cell parameters at different temperatures

A single-crystal X-ray diffraction studies revealed that CaDMP crystallizes from water solution as a 1D coordination polymer in a monoclinic space group *P*2_1_/*c* – the crystal data and experimental conditions are summarized in Table S2, S3 and S4 (SI).

**Fig. 1:**
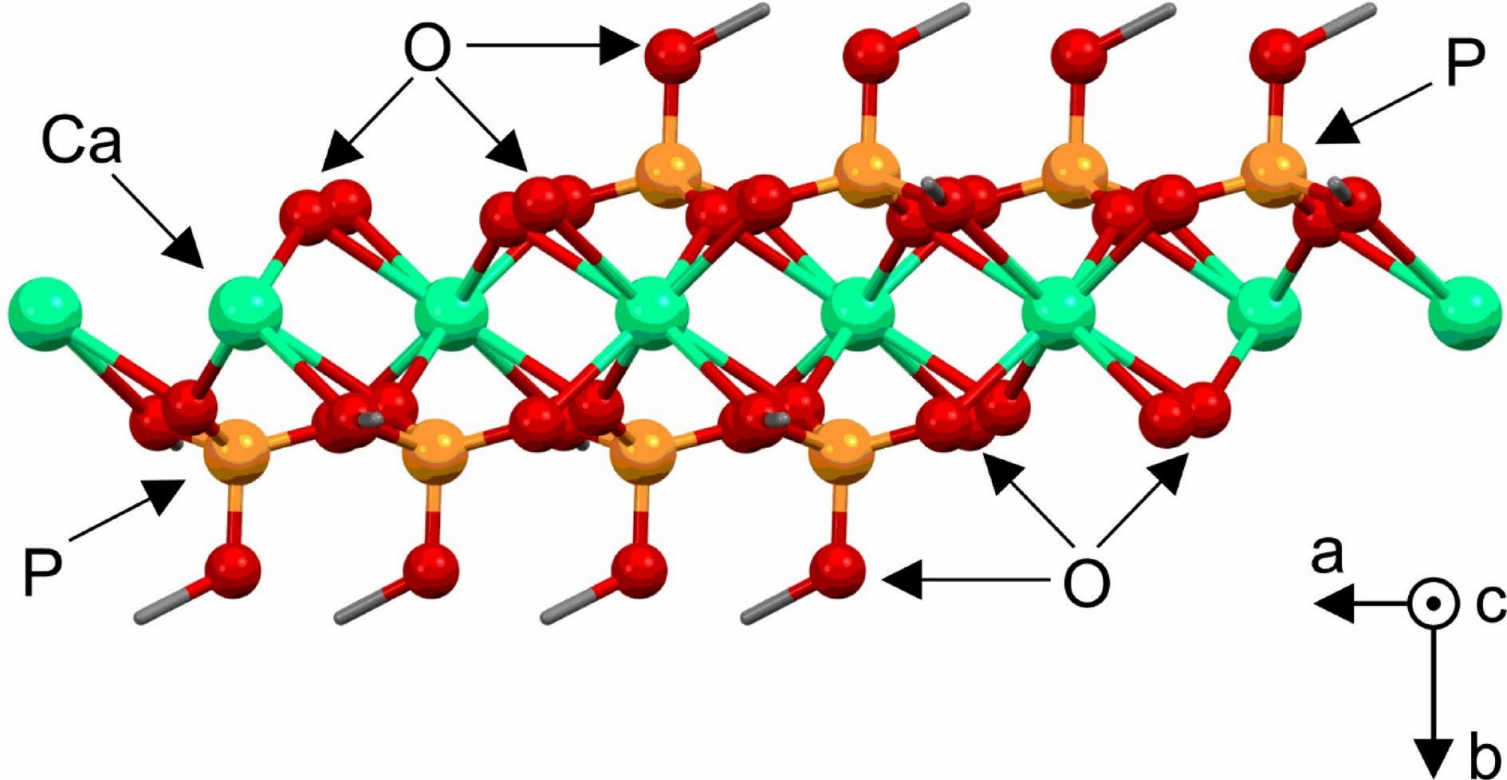
Structure of the 1D polymeric chain of CaDMP in a view along the crystallographic *z*-axis. For the clarity of presentation hydrogen and carbon atoms are omitted.

The asymmetric unit of CaDMP, comprising one calcium cation and two dimethylphosphate (DMP) ligands, is depicted in Fig. S8 (SI). In the crystal structure of CaDMP calcium cations are surrounded by six DMP moieties occupying eight coordination sites of the metal center (Fig. S8 in SI). Each Ca^2+^ ion is linked with the adjacent one via a bridging binding mode of the tridentate DMP ligand thus forming a 1D polymer, as shown in Fig. 1. One methoxy group of each DMP anion remains uncoordinated and acts as a terminal group isolating the neighbouring polymeric chains (Fig. S9 in SI). SEM images showed that 1D polymeric chains of CaDMP organized into a few micrometre long, rod-like particles having a hexagonal cross-section (Fig. S10 in SI).

In accordance with the convention recommended by IUPAC^82^, the methyl substituents around both crystallographically distinct phosphorus centers within CaDMP structure adopt a thermodynamically stable *gauche*–*gauche* conformation (*G*^+^*G*^+^)^78^ since their dihedral angles are within the 65–83° and 62–70° regions. The analysis by means of the bond–valence vector (BVV) model^83^ (see SI for a detailed description of its protocol) indicated almost no structural strains in the geometry of calcium and phosphorus coordination polyhedra, since the values of the length of the resultant BVV calculated for them were on a very low level (ca. 0.04 and 0.10 valence units for phosphorus and calcium, respectively, Table S6 in SI). These structural features were enough to create crystallographically distinct PO_4_ tetrahedra, that were, however, indistinguishable from one another in the ^31^P MAS NMR spectrum of CaDMP (see Fig. S2 in SI).

It should be noted, that the structural analysis of CaDMP was carried out at −153 °C, which was significantly lower than the temperature usually applied during β-iPP crystallization (ca. 120–140 °C). A knowledge that all materials are prone to temperature-driven structural transformations (e.g., thermal expansion/contraction and/or polymorphic transitions), combined with the fact that a successful epitaxial growth of the β-iPP phase on the surface of NAs particles requires a good matching between their crystal lattice parameters^23,36,47^, prompted us to investigate the impact of temperature on the CaDMP structure. DSC curves recorded for CaDMP between −150 and 220 °C (Fig. S11 in SI) did not contain any signals attributable to thermal effects of the first- or second-order phase transitions suggesting that, within this temperature region, CaDMP remained solid and existed in a single polymorphic form. The same conclusions could be derived from the PXRD diffractograms recorded at different temperatures (i.e., the results of a variable temperature PXRD, VT-PXRD, measurements) – Fig. S12 and Table S7 in SI contain data on the CaDMP crystal lattice parameters obtained by means of a careful indexing of the observed X-ray reflections. As can be seen, the unit cell of CaDMP underwent a typical expansion on heating from room temperature to 190 °C (or contraction when being cooled from that temperature), albeit this process was characterized by a large anisotropy. The susceptibility of the CaDMP lattice parameters to the temperature changes increased in the order of *a*- < *c*- < *b*-parameter, as shown by the values of the linear thermal expansion coefficients (α_*i*_) along the chosen crystallographic *i* direction calculated according to the previously described method^84^: for the VT-PXRD heating mode α_*a*_, α_*b*_ and α_*c*_ equalled to 5.84(92) ×10^–6^, 11.41(20) ×10^–5^ and 2.46(9) ×10^–5^K^–1^, respectively, whereas the volumetric thermal expansion coefficient β_*V*_ had the value of 14.51(29) ×10^–5^ K^–1^ (see Table S8 in SI). It should be noted that the values of CaDMP lattice parameters and unit cell volume calculated at −153 °C using Eqs. (S10)–(S13) (SI), were very close to those directly determined for CaDMP monocrystal (in each case the difference was less than 1% of the experimental value), which not only proved the correctness of these equations but was also a clear evidence that within the investigated temperature region (e.g., between −153 and 190 °C) CaDMP did not reorganize its crystal structure.

## iPP-nucleating properties of CaDMP

### Isothermal crystallization

**Fig. 2:**
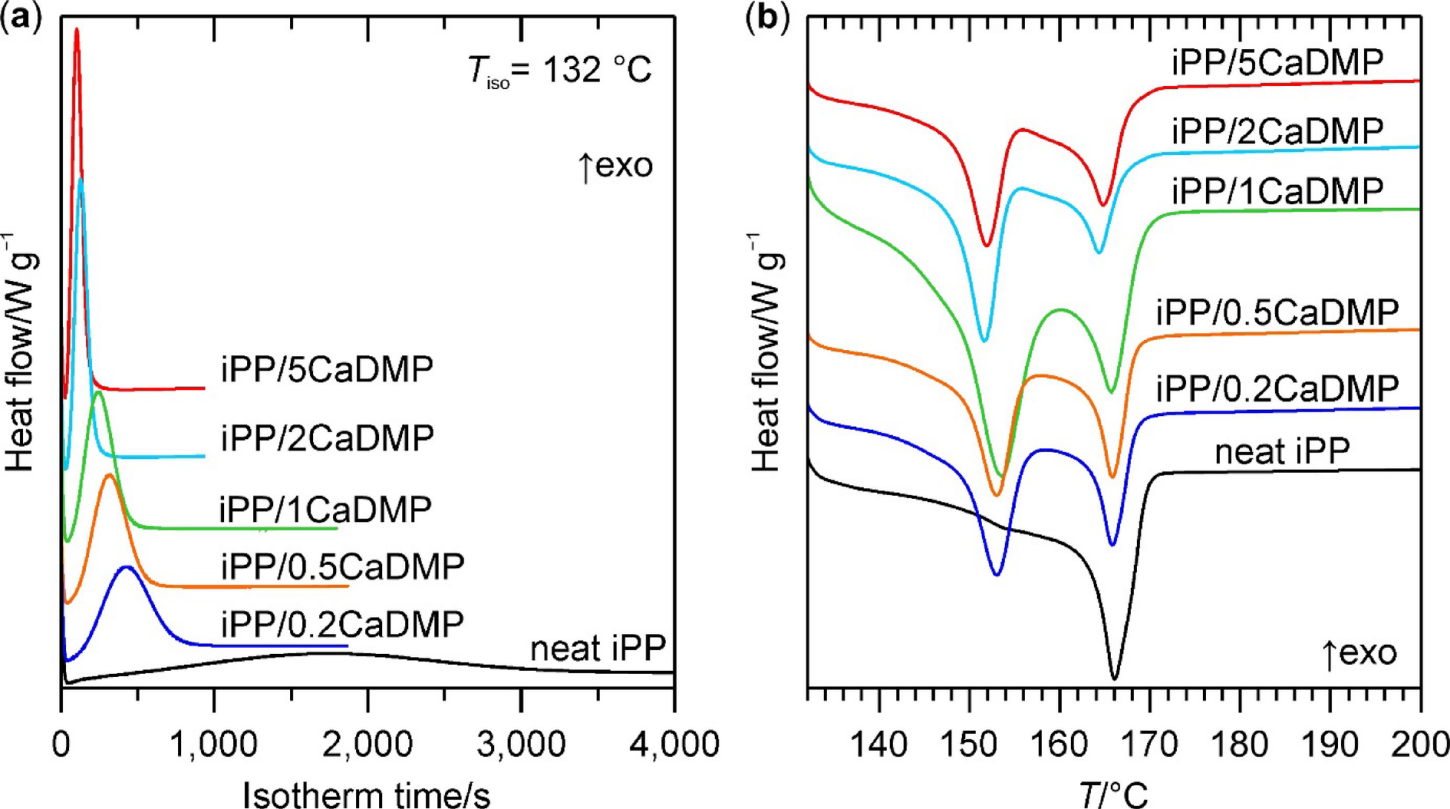
DSC curves of neat iPP and its composites with different loadings of CaDMP recorded during: (**a**) an isotherm at 132 °C, and (**b**) a subsequent heating to 200 °C (with a heating rate of 10 °C min^–1^).

The impact of CaDMP on the isothermal crystallization of iPP was investigated at 132 °C for the samples of neat iPP and its composites loaded with 0.2–5.0 wt% of the filler. Figure 2; Table 1 present the DSC data on the evolution of crystallization exotherms during an isothermal cycle, as well as a melting behaviour of the thus crystallized iPP phases. Abbreviations: Δ*H*_c, total_ – total enthalpy of crystallization (an overall area of the crystallization exotherm) calculated with respect to the mass of polymer in the sample; *T*_m1_ and *T*_m2_ – melting temperatures recorded during heating of the previously crystallized samples; $\:{X}_{\text{c},\text{m}}^{\text{t}\text{o}\text{t}\text{a}\text{l}}$ – overall absolute degree of crystallinity of the sample calculated based on the melting endotherms (see a detailed description in SI); $\:{k}_{\beta\:}^{\text{D}\text{S}\text{C}}$ – fraction of the β-iPP crystals within the iPP crystal phase determined by means of DSC.

**Table 1:** Selected DSC parameters estimated during isothermal crystallization carried out at 132 °C for neat iPP and its composites with cadmp.

CaDMP content/wt%	ΔH_c, total_/J g^–1^	$\:{t}_{\text{ind}}$/s	$\:{t}_{\text{c}}^{\text{p}}$/s	$\:{t}_{\text{c}}^{\text{F}\text{W}\text{H}\text{M}}$/s	T_m1_/°C	T_m2_/°C	$\:{X}_{\text{c},\text{m}}^{\text{t}\text{o}\text{t}\text{a}\text{l}}$/%	$\:{k}_{\beta\:}^{\text{D}\text{S}\text{C}}$
	*isothermal step*				*heating step after* *isothermal crystallization*			
0.0	95.96	481	1,742	1,599.7	–	166.07	61.1	–
0.2	80.19	155	427	331.2	152.97	165.76	57.1	0.56
0.5	81.97	114	315	237.6	153.01	165.79	57.9	0.52
1.0	83.81	78	243	198.7	153.65	165.75	56.8	0.52
2.0	79.30	46	128	89.8	151.63	164.32	53.1	0.57
5.0	82.88	34	102	72.2	151.94	164.82	56.2	0.47

As can be seen in Fig. 2, the isothermal crystallization of neat iPP at 132 °C was a time-consuming process and required more than 1 h for its completion. Incorporating CaDMP particles into iPP substantially increased the rate at which the latter crystalized, since with increasing CaDMP content the crystallization exotherm became narrower and moved to the lower values region on the time axis. This observation was also supported by the changes in the values of all time parameters usually determined for the isothermal crystallization of polymers, namely the induction time (a period of time from the beginning of the isotherm to the first deviation of the DSC signal from the baseline, *t*_ind_), time of the exotherm maximum ($\:{t}_{\text{c}}^{\text{p}}$), and the crystallization exotherm’s full width at half maximum ($\:{t}_{\text{c}}^{\text{F}\text{W}\text{H}\text{M}}$). From the data presented in Table 1 it is evident that with increasing content of CaDMP the values of all these parameters were lowered compared to neat iPP (by 68–96% depending on both the type of a parameter and the filler’s loading in the composite). The best effects were observed for the system with the highest content (i.e., 5 wt%) of CaDMP, in which both the nucleation stage of the crystallization process was accelerated (e.g., its *t*_ind_ was lowered to 34 s from 481 s characterizing the unmodified iPP matrix), and the overall rate of the iPP crystal phase growth increased (e.g., ca. 22 times reduction in its $\:{t}_{\text{c}}^{\text{F}\text{W}\text{H}\text{M}}$ in comparison to neat iPP).

In order to carry out a more detailed analysis of the iPP isothermal crystallization kinetics we applied a widely-recognized mathematical modeling method based on the Avrami–Evans theory that relates the relative volumetric degree of crystallinity of the sample (*X*_t_) to the time elapsed since the beginning of the crystallization exotherm formation^85^. By taking into account the overall time of the DSC isothermal cycle (*t*) and *t*_ind_, the basic and simplest form of the Avrami–Evans equation can be expressed by Eq. (1)^85^:

1 $$\:1-{X}_{\text{t}}={\text{e}}^{-{k}_{\text{A}\text{v}\text{r}\text{a}\text{m}\text{i}}{(t-{t}_{\text{i}\text{n}\text{d}})}^{{n}_{\text{A}\text{v}\text{r}\text{a}\text{m}\text{i}}}}$$

or its linearized (double logarithmic) version (Eq. (2)):

2 $$\:\text{l}\text{n}[-\text{l}\text{n}\left[1-{X}_{\text{t}}\right]=\text{l}\text{n}\left({k}_{\text{A}\text{v}\text{r}\text{a}\text{m}\text{i}}\right)+{n}_{\text{A}\text{v}\text{r}\text{a}\text{m}\text{i}}\text{l}\text{n}\left(t-{t}_{ind}\right)$$

where *n*_Avrami_ is the Avrami index related to the dimensionality of the growing crystals and nucleation mode, whereas *k*_Avrami_ denotes the Avrami overall crystallization rate constant referring to both nucleation and crystal growth stages. It should be noted that *k*_Avrami_ is given in units of $\:{\text{t}\text{i}\text{m}\text{e}}^{-{n}_{\text{A}\text{v}\text{r}\text{a}\text{m}\text{i}}}$ and for comparison purposes should be normalized to the *n*_Avrami_–independent parameter *K*_Av_ (expressed in units of time^–1^) by elevating its value to the power *n*_Avrami_^−1^ (i.e., $\:{k}_{\text{A}\text{v}\text{r}\text{a}\text{m}\text{i}}={{K}_{\text{A}\text{v}}}^{{n}_{\text{A}\text{v}\text{r}\text{a}\text{m}\text{i}}}$). Moreover, for the known values of *n*_Avrami_, *k*_Avrami_ and *X*_t_= 0.5 a solution of Eq. (1) gives Eq. (3) defining the theoretical half crystallization time ($\:{t}_{0.5}^{\text{A}\text{v}\text{r}\text{a}\text{m}\text{i}}$)^85^.

3 $$\:{t}_{0.5}^{\text{A}\text{v}\text{r}\text{a}\text{m}\text{i}}={\left(\frac{\text{l}\text{n}2}{{k}_{\text{A}\text{v}\text{r}\text{a}\text{m}\text{i}}}\right)}^{\raisebox{1ex}{$1$}\!\left/\:\!\raisebox{-1ex}{${n}_{\text{A}\text{v}\text{r}\text{a}\text{m}\text{i}}$}\right.}$$

**Fig. 3:**
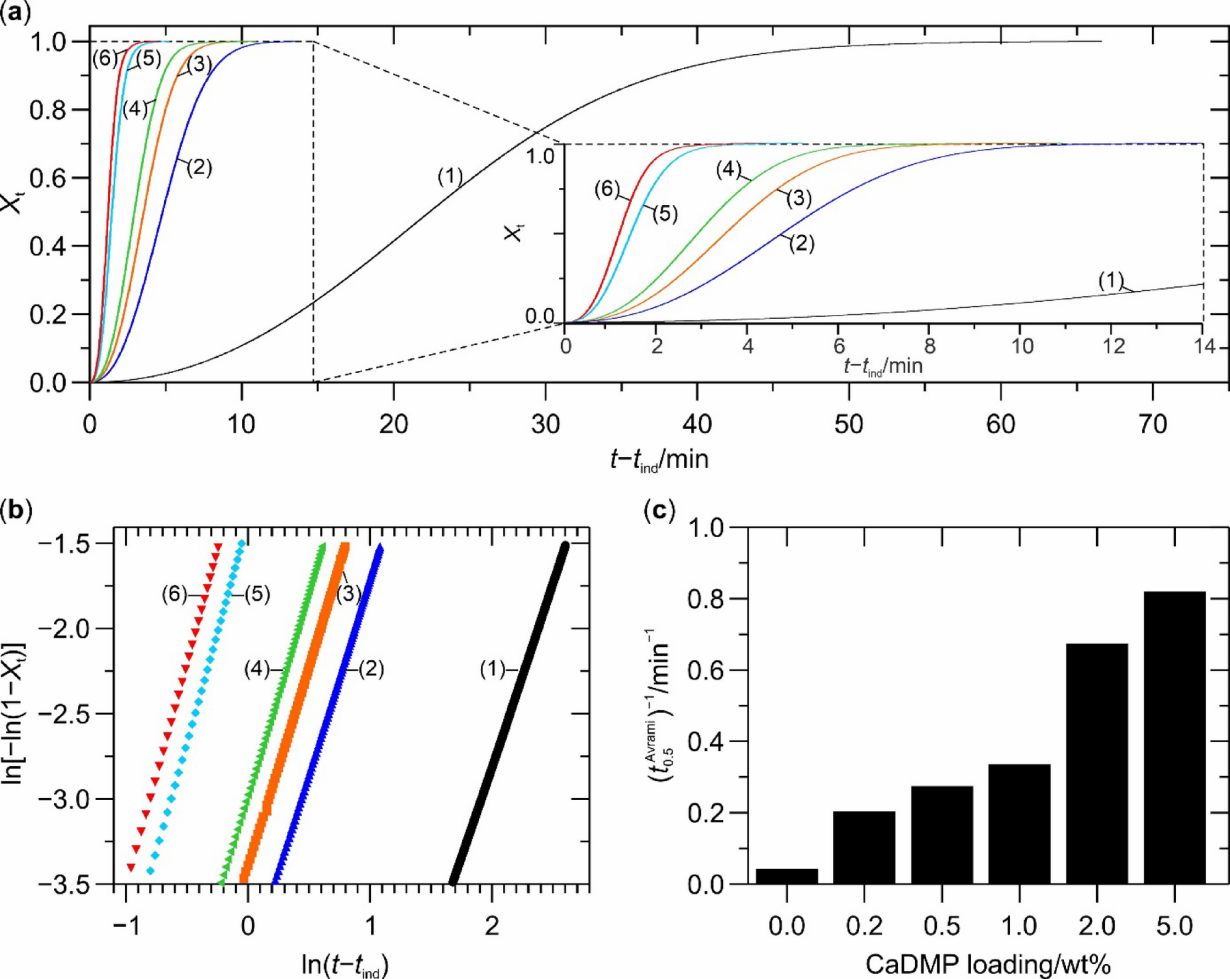
Kinetics of the isothermal crystallization of neat iPP and its composites with various CaDMP loadings: (**a**) variation of *X*_t_ with isothermal crystallization time, (**b**) Avrami plots from which *n*_Avrami_ and *k*_Avrami_ were obtained, (**c**) changes in the overall crystallization rate (reciprocal crystallization half time). (1) neat iPP; (2) iPP/0.2CaDMP; (3) iPP/0.5CaDMP; (4) iPP/1CaDMP; (5) iPP/2CaDMP; (6) iPP/5CaDMP.

In the case of neat iPP and its composites with CaDMP we calculated the *X*_t_ values at various periods of isothermal crystallization process by integrating their DSC exotherms and subsequently applying Eq. (4):

4 $$\:{X}_{\text{t}}={\int\:}_{{t}_{\text{i}\text{n}\text{d}}}^{t}\left(\frac{{\text{d}H}_{\text{c}}}{\text{d}t}\right)\text{d}t/{\int\:}_{{t}_{\text{i}\text{n}\text{d}}}^{{t}_{{\infty\:}}}\left(\frac{{\text{d}H}_{\text{c}}}{\text{d}t}\right)\text{d}t$$

where d*H*_c_ refers to the measured enthalpy of crystallization during an infinitesimal period of time *dt* and *t*_∞_ is the time required for the completion of the crystallization process. By plotting ln[−ln(1 − *X*_t_)] versus ln(*t* − *t*_ind_) we obtained the values of both *n*_Avrami_ and ln(*k*_Avrami_) as the slopes and intercepts of the resulting straight lines, respectively. Figure 3 shows the variation of *X*_t_ with the time of isothermal crystallization and the Avrami plots applied in the linear fitting procedure, whereas Table 2 contains the Avrami kinetic parameters calculated for the investigated polymeric systems (*R*^2^ is the coefficient of determination providing information about the goodness of fit of the Avrami linear regression model, $\:{t}_{0.5}^{\text{A}\text{v}\text{r}\text{a}\text{m}\text{i}}$ and $\:{t}_{0.5}^{\text{e}\text{x}\text{p}}$ denote crystallization half time calculated based on the Avrami–Evans theory in accordance to Eq. 3 and estimated from the experimental datapoints presented in Fig. 3, respectively, rounded to the nearest integer). It should be noted that, in accordance with the guidelines proposed in the literature^86^, the linear fitting was carried out only for the data corresponding to the *X*_t_ values between 0.03 and 0.20, since it minimized errors related to either a low value of the heat flow signal at the initial period of the iPP isothermal crystallization, or a nonlinearity of the Avrami plots occurring during the secondary crystallization stage.

**Table 2:** Kinetic parameters calculated based on the Avrami-Evans theory applied to isothermal crystallization (*T =* 132 °C) of neat iPP and its composites with cadmp.

CaDMP loading/wt%	*n*_Avrami_	k_Avrami_/$\:{\text{m}\text{i}\text{n}}^{{-n}_{\text{A}\text{v}\text{r}\text{a}\text{m}\text{i}}}$	*R* ^2^	K_Av_ /min^–1^	$\:{t}_{0.5}^{\text{A}\text{v}\text{r}\text{a}\text{m}\text{i}}/\text{s}$	$\:{t}_{\text{0,5}}^{exp}/\text{s}$
0.0	2.144(1)	8.23(2) ×10^–4^	0.99991	0.0364(1)	1,389	1,342
0.2	2.285(2)	0.01831(3)	0.99993	0.1736(4)	294	287
0.5	2.351(3)	0.03336(5)	0.99991	0.2355(6)	218	213
1.0	2.385(3)	0.05111(5)	0.99993	0.2874(5)	179	176
2.0	2.575(5)	0.2518(6)	0.99989	0.5854(11)	89	88
5.0	2.636(5)	0.4107(12)	0.99992	0.7135(13)	73	72

Analysis of the *X*_t_ versus (*t* − *t*_ind_) curves (Fig. 3) clearly indicated that with increasing concentration of CaDMP within the iPP composite the system required less time to reach any given value of *X*_t_, thus suggesting that the overall rate of crystallization was also increased. This phenomenon could be quantified by tracking the changes in the values of two important kinetic parameters: the already mentioned *k*_Avrami_ (or its *n*_Avrami_-independent alternative, *K*_Av_), as well as crystallization half time (a time that elapsed from the onset of crystallization exotherm until the relative crystallinity of the system reached half of its total level) whose inverse represents the overall rate of isothermal crystallization of iPP^85^. The data presented in Table 2; Fig. 3 show that even the smallest amount of CaDMP (i.e., 0.2 wt%) caused a 22-times increase in *k*_Avrami_ (which equals to an almost fivefold increase in *K*_Av_) and reduced the value of $\:{t}_{0.5}^{\text{A}\text{v}\text{r}\text{a}\text{m}\text{i}}$ by ca. 79% compared to neat iPP. This general trend continued for the other composite systems, so that the one containing 5 wt% CaDMP exhibited the *k*_Avrami_, *K*_Av_ and $\:{t}_{0.5}^{\text{A}\text{v}\text{r}\text{a}\text{m}\text{i}}$ values of 0.4107(12) $\:{\text{m}\text{i}\text{n}}^{{-n}_{\text{A}\text{v}\text{r}\text{a}\text{m}\text{i}}}$, 0.7135(13) min^–1^ and 73 s, respectively. For that composite the overall rate of isothermal crystallization equalled ca. 0.82 min^–1^, whereas at the same temperature conditions the neat iPP sample crystallized with a rate of ca. 0.04 min^–1^ (Fig. 3). It should be noted, that the differences between the values of half crystallization times calculated from the Avrami equations ($\:{t}_{0.5}^{\text{A}\text{v}\text{r}\text{a}\text{m}\text{i}}$) and those estimated from the experimental data ($\:{t}_{0.5}^{\text{e}\text{x}\text{p}}$) were very small (ca. 1.1–3.5% of the $\:{t}_{0.5}^{\text{e}\text{x}\text{p}}$values), which proved a high goodness of fit of the adopted mathematical model^85^.

Interestingly, the incorporation of CaDMP into iPP was accompanied by a small (ca. 13–17%) decrease in the total enthalpy of isothermal crystallization (see Table 1). In our opinion, this phenomenon could be explained in terms of a lower absolute crystallinity of the composite samples, and/or the changes in the polymorphic type of iPP crystal phases that were formed therein. In fact, in the case of the iPP/CaDMP samples the DSC heating curves recorded directly after their isothermal crystallization (see Fig. 2) showed the occurrence of two partially overlapping endothermal transitions located around 152 and 165 °C, contrary to the neat iPP sample that underwent only one melting process at ca. 166 °C (Table 1). Based on the literature data^2^, the process proceeding at higher temperature can be ascribed to the melting of α-iPP, whereas that occurring at lower temperature – to the melting of β-iPP. Thus, one could conclude that the neat iPP sample contained only the former polymorph, whereas the iPP/CaDMP composites were a mixture of the α- and β-iPP crystal phases. Based on the DSC procedure proposed in the literature^46^, we approximated the contribution of β-iPP to the overall absolute degree of iPP crystallinity ($\:{X}_{\text{c},\text{m}}^{\text{t}\text{o}\text{t}\text{a}\text{l}}$) of the investigated materials (β-iPP fraction, $\:{k}_{\beta\:}^{\text{D}\text{S}\text{C}}$) defined by Eq. 5:

5 $$\:{k}_{\beta\:}^{\text{D}\text{S}\text{C}}=\frac{{X}_{\text{c},\text{m}}^{\beta\:}}{{X}_{\text{c},\text{m}}^{\text{t}\text{o}\text{t}\text{a}\text{l}}}=\frac{{X}_{\text{c},\text{m}}^{\beta\:}}{{X}_{\text{c},\text{m}}^{\alpha\:}+{X}_{\text{c},\text{m}}^{\beta\:}}$$

where $\:{X}_{\text{c},\text{m}}^{\alpha\:}$ and $\:{X}_{\text{c},\text{m}}^{\beta\:}$ are the relative crystallinities of the α- and β-iPP phase, respectively (for a more detailed description of this procedure see Fig. S13 in SI). The obtained values of $\:{X}_{\text{c},\text{m}}^{\text{t}\text{o}\text{t}\text{a}\text{l}}$ and $\:{k}_{\beta\:}^{\text{D}\text{S}\text{C}}$ (Table 1) suggest that the incorporation of CaDMP into iPP had a negligible effect on the overall ability of iPP to formation of the crystalline domains, since the $\:{X}_{\text{c},\text{m}}^{\text{t}\text{o}\text{t}\text{a}\text{l}}$ values of the iPP/CaDMP composites were on a comparable level with that of the unmodified iPP. However, the presence of CaDMP particles caused that approximately half of the obtained polymer crystals exhibited the β-iPP structure.

**Fig. 4:**
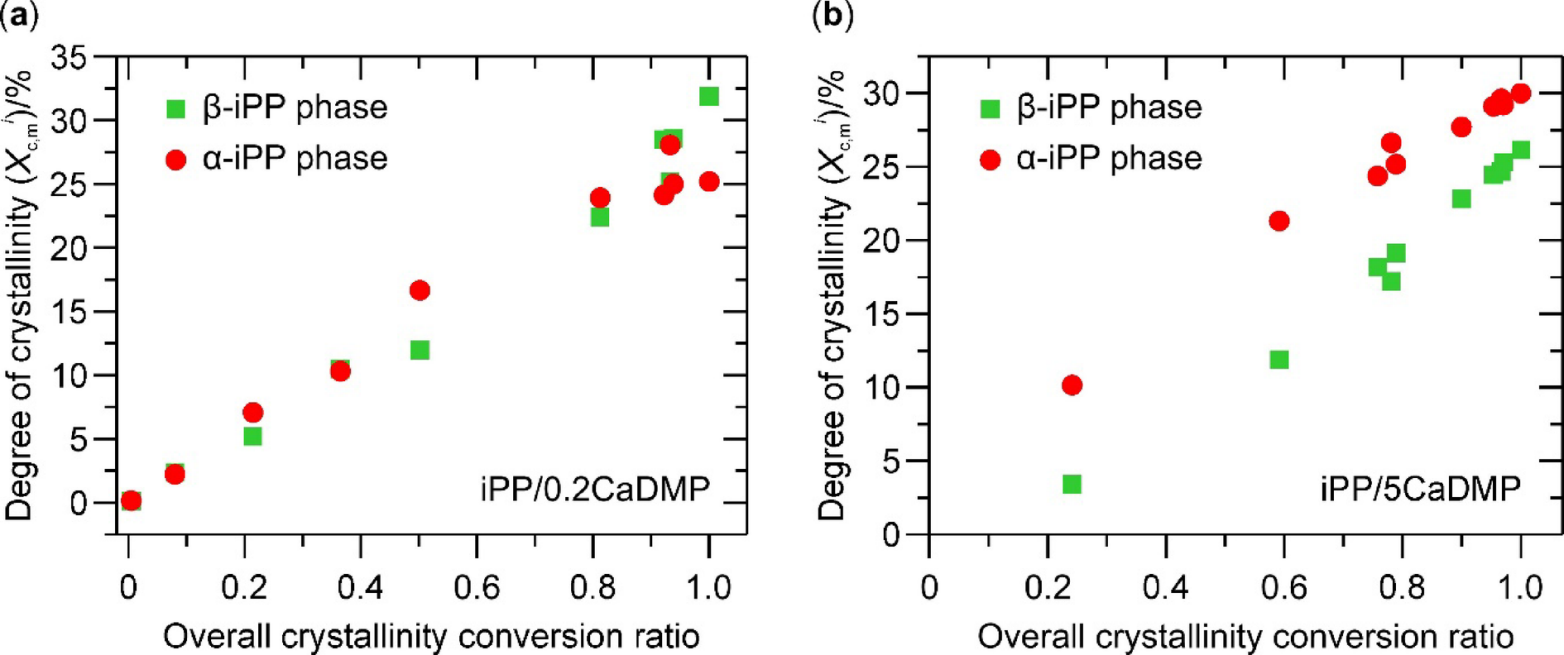
Isothermal stepwise crystallization. Variation of the α-iPP and β-iPP contents (expressed as a degree of crystallinity of a given crystal phase *i*, $\:{X}_{\text{c},\text{m}}^{i}$) with a progress of the overall crystallization in the iPP-based composites with: (**a**) 0.2 wt% and (**b**) 5 wt% of CaDMP.

It is worth noting, that the kinetics of isothermal crystallization of the α- and β-iPP phases cannot be evaluated separately during a conventional calorimetric experiment due to a lack of separation of their respective exotherms. In order to overcome this problem, we applied the isothermal stepwise crystallization (ISC) procedure^85^. The samples had been isothermally crystallized for different periods of time to attain different degrees of crystallinity, and then their polymorphic compositions were investigated from the melting endotherms recorded during a subsequent heating steps. The results of these experiments for the iPP/0.2CaDMP and iPP/5CaDMP composites are shown in Fig. 4. Based on these data, one can conclude that regardless of the CaDMP content both polymorphs of iPP were formed simultaneously and could be detected from the beginning of the isothermal crystallization process. However, in the case of the system with the lowest NA loading the content of each iPP polymorph increased in a comparable manner, with β-iPP dominating the crystal domains only at the highest crystallinity conversion. On the other hand, in iPP/5CaDMP α-iPP prevailed over β-iPP throughout the entire process, albeit the difference between their contents diminished with the progress of isothermal crystallization. The reason for such behavior is still unknown and needs further experimental investigation.

### Crystallization under non-isothermal conditions

**Fig. 5:**
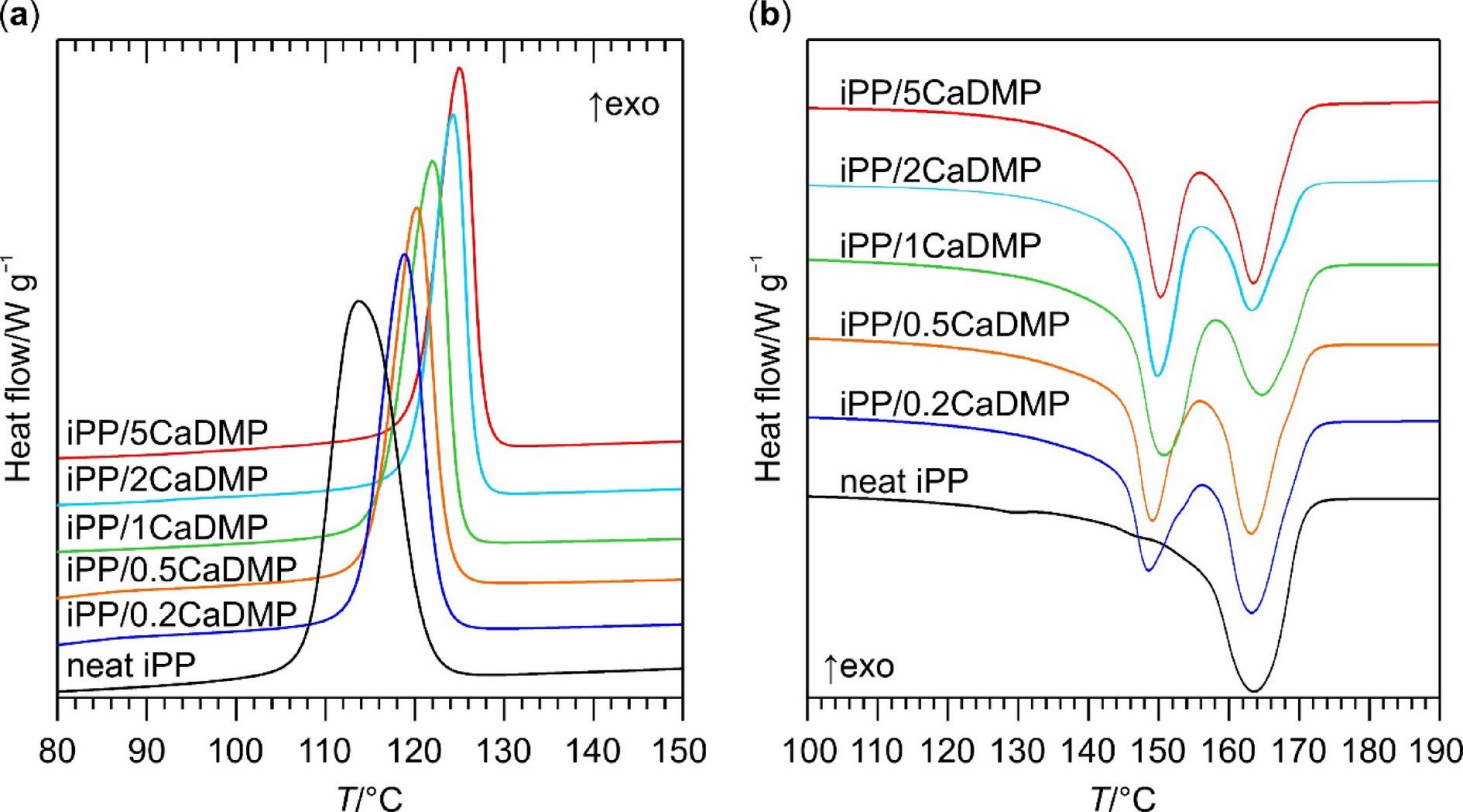
Normalized DSC traces of neat iPP and its composites with different loadings of CaDMP recorded during: (**a**) a cooling cycle, and (**b**) a subsequent heating to 190 °C. Temperature change of 10 °C min. For the clarity of presentation only the temperature regions corresponding to the iPP crystallization or melting are shown.

The process of crystallization under non-isothermal conditions was investigated for neat iPP and its composites with CaDMP by means of DSC during cooling from the melt (at a cooling rate of 10 °C min^–1^). The type of the resulting crystal domains was analysed the same way as in the case of isothermal crystallization, i.e., thru their melting endotherms occurring during a subsequent heating cycle. Figure 5 presents the DSC curves recorded during both these cycles, while some of the characteristic parameters of crystallization are summarized in Table 3: *T*_ini_ – initial temperature of crystallization; $\:{T}_{\text{c}}^{\text{p}}$ and $\:{T}_{\text{c}}^{\text{F}\text{W}\text{H}\text{M}}$– maximum and full width at half maximum of the exothermic peak, respectively; $\:{t}_{0.5}^{\text{e}\text{x}\text{p}}$ – crystallization half time estimated from the experimental datapoints (its value rounded to the nearest integer); $\:{X}_{\text{c},\text{m}}^{\text{t}\text{o}\text{t}\text{a}\text{l}}$ – absolute degree of crystallinity of the sample calculated based on the melting endotherms; $\:{k}_{\beta\:}^{\text{D}\text{S}\text{C}}$ – fraction of the β-iPP crystals within the iPP crystal phase.

**Table 3:** Selected parameters estimated during non-isothermal crystallization of the iPP/CaDMP composites carried out at cooling/heating rates of 10 °C min^–1^.

CaDMP content/wt%	ΔH_c, total_ /J g	$\:{T}_{\text{i}\text{n}\text{i}}$/°C	$\:{T}_{\text{c}}^{\text{p}}$/°C	$\:{T}_{\text{c}}^{\text{F}\text{W}\text{H}\text{M}}$/°C	$\:{t}_{\text{0,5}}^{\text{e}\text{x}\text{p}}$/s	T_m1_/°C	T_m2_/°C	$\:{X}_{\text{c},\text{m}}^{\text{t}\text{o}\text{t}\text{a}\text{l}}$/%	$\:{k}_{\beta\:}^{\text{D}\text{S}\text{C}}$
0.0	94.70	127.47	113.74	8.24	79	–	163.57	58.4	–
0.2	91.79	129.09	118.78	5.22	64	148.60	163.55	58.6	0.37
0.5	93.33	130.59	120.28	4.96	66	149.06	163.38	59.4	0.43
1.0	90.20	128.48	121.91	5.16	45	150.76	164.69	60.5	0.54
2.0	91.77	129.59	124.27	4.10	36	149.71	163.16	58.8	0.54
5.0	98.45	130.92	124.91	4.49	41	150.28	163.47	59.7	0.43

In comparison with neat iPP, the crystallization exotherms of the systems filled with CaDMP became narrower and were shifted towards higher temperatures. Based on these findings, one can assume that under the investigated non-static thermal conditions a crystallization of the iPP matrix was facilitated upon incorporation of the CaDMP particles: a small (i.e. by ca. 1–4 °C) increase in the *T*_ini_ values indicated slightly lower energy barriers during the nucleation stage, while the higher temperatures of the maximum heat release rate ($\:{T}_{\text{c}}^{\text{p}}$) combined with a 36–50% decrease in the $\:{T}_{\text{c}}^{\text{F}\text{W}\text{H}\text{M}}$ values could result from a larger number of crystallization nuclei being formed and/or the positively affected crystal growth rate of iPP. A combination of both these effects effectively accelerated the iPP crystal phase formation, since even at the lowest filler’s concentration (i.e. 0.2 wt% of CaDMP) the overall crystallization rate (approximated by the reciprocal crystallization half time) was increased by ca. 34% compared to the unmodified iPP. The extent of this change became even greater at higher CaDMP loadings, reaching a level of the 83–93% increase in the case of the samples containing 2–5 wt% of CaDMP. It is worth noting, that the presence of CaDMP during cooling of the molten iPP matrix promoted a formation of the α- and β-iPP crystals, as revealed by the bimodal melting profiles of the investigated composites (see Fig. 5). In fact, the recorded DSC melting curves, as well as crystallinity of the samples and the contents of each iPP polymorph (see $\:{X}_{\text{c},\text{m}}^{\text{t}\text{o}\text{t}\text{a}\text{l}}$ and $\:{k}_{{\upbeta\:}}^{\text{D}\text{S}\text{C}}$ values presented in Table 3) are close to those estimated during a previously discussed process of isothermal crystallization.

The results of preliminary studies discussed above, prompted us to a more detailed investigation of the impact of CaDMP on the kinetics of non-isothermal crystallization of iPP. For that purpose, neat iPP and its two composites (i.e. the systems filled with 0.2 wt% and 1.0 wt% of CaDMP) were selected and crystallized from melt at different cooling rates (*φ*, 3–10 °C min^–1^). The recorded crystallization exotherms were analyzed and integrated (see Table 4 and Fig. S14 in SI), providing the data for a calculation of the relative crystallinity as a function of temperature (*X*_*T*_) according to Eq. (6):

**Table 4:** Selected parameters estimated during non-isothermal crystallization of iPP and its composites filled with 0.2 or 1.0 Wt% of CaDMP carried out at different cooling rates.

CaDMP content/wt%	φ/°C min^–1^	ΔH_c, total_/J g^–1^	$\:{T}_{\text{i}\text{n}\text{i}}$/°C	$\:{T}_{\text{c}}^{\text{p}}$/°C	$\:{T}_{\text{c}}^{\text{F}\text{W}\text{H}\text{M}}$/°C	$\:{t}_{\text{0,5}}^{\text{e}\text{x}\text{p}}$/s	CRP/°C^–1^
0.0	3	97.28	131.94	118.55	5.50	249	0.066(18)
	5	94.05	125.49	116.29	3.85	112	
	7	94.75	128.03	113.71	7.36	116	
	10	94.70	127.47	113.74	8.24	79	
0.2	3	92.85	133.19	124.71	3.60	166	0.083(16)
	5	90.90	130.89	122.32	4.10	103	
	7	92.80	128.96	120.67	4.73	72	
	10	91.79	129.09	118.78	5.22	64	
1.0	3	92.59	133.71	127.01	3.37	139	0.130(6)
	5	91.26	131.14	124.58	3.42	81	
	7	90.82	130.36	123.98	4.20	60	
	10	90.20	128.48	121.91	5.16	45	

6 $$\:{X}_{T}={\int\:}_{{T}_{\text{i}\text{n}\text{i}}}^{{T}_{\text{c}}}\left(\frac{{\text{d}H}_{\text{c}}}{\text{d}T}\right)\text{d}T/{\int\:}_{{T}_{\text{i}\text{n}\text{i}}}^{{T}_{\infty\:}}\left(\frac{{\text{d}H}_{\text{c}}}{\text{d}T}\right)\text{d}T$$

where *T*_c_ or *T*_∞_ refer to the crystallization temperature at the time *t* or after the completion of the non-isothermal crystallization process, respectively. *X*_*T*_ can be converted to a time-resolved *X*_*t*_ by applying Eq. (7). Fig. S14 (SI) presents the evolution of *X*_*t*_ with the non-isothermal crystallization time *t* for 3 materials mentioned above.

7 $$\:t=\frac{\left|{T}_{\text{i}\text{n}\text{i}}-{T}_{\text{c}}\right|}{\phi\:}$$

As can be seen in Table 4 for all tested materials, an increase in a cooling rate led to a shift of the crystallization peak to the lower temperature region, and its widening. At any given cooling rate, the estimated temperatures of the initiation and maximum rate of crystallization characterizing the systems modified with CaDMP were higher than those related to neat iPP, and this trend intensified with the increasing CaDMP content. All these findings proved that the crystallization of iPP accelerated in the presence of CaDMP, regardless of the rate of temperature change. In order to evaluate this more quantitatively, we analyzed the crystallization rate parameter (CRP), which can be considered as a factor representing the overall crystallization rate during a non-isothermal crystallization process. CRP was first introduced for polypropylene by Ma and coworkers, and defined as the slope of a straight line obtained after plotting the reciprocal of crystallization half time versus *φ*^87^. Our composite systems loaded with 0.2 or 1.0 wt% of CaDMP exhibited CRP values higher by ca. 45% or 85%, respectively, than that of the unmodified iPP, indicating a much faster crystallization (see Table 4, standard error of the CRP value is presented in parenthesis). This conclusion found further support in the results of a mathematical modeling of the non-isothermal crystallization kinetics according to the Liu and Mo methodology^88^.

**Fig. 6:**
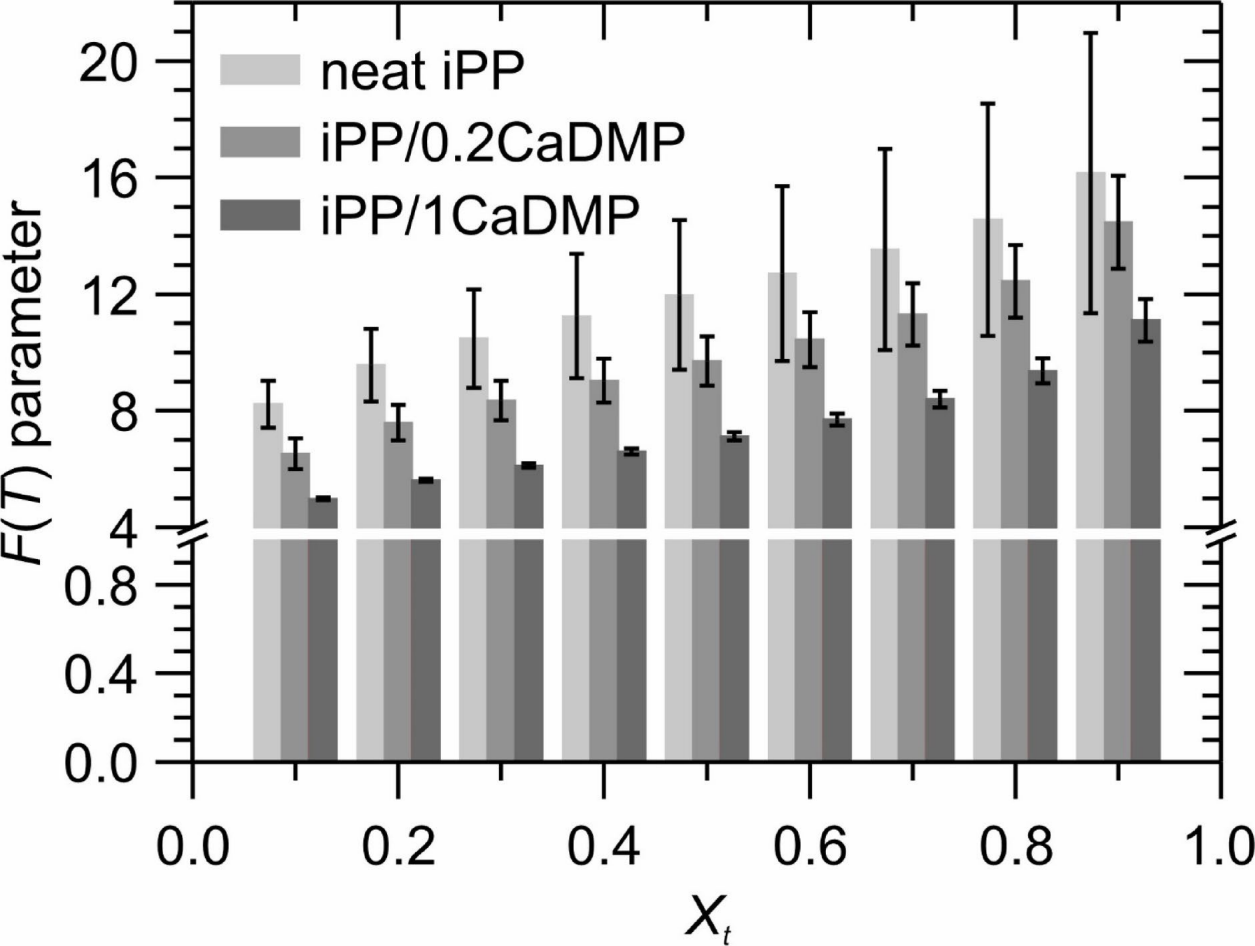
Non-isothermal crystallization of iPP and its composites with CaDMP: variation of the Liu and Mo kinetic parameter *F*(*T*) with *X*_*t*_.

At a given relative degree of crystallinity, the Liu and Mo model correlates the crystallization time *t* and the cooling rate *φ*, following a simplified Eq. (8):

8 $$\:\text{l}\text{n}\left|\phi\:\right|=\text{l}\text{n}F\left(T\right)-a\text{l}\text{n}t$$

where *a* is the parameter containing information about the dimensionality of crystallization and nucleation mode^88^. At a given *X*_*t*_, the $\:\text{l}\text{n}\left|\phi\:\right|$ versus $\:\text{l}\text{n}t$ plot produces a straight line and the values of *a* and ln*F(T)* can be easily obtained as the slope and intercept of that line, respectively. *F*(*T*) is a temperature-dependent complex function that refers to the cooling rate value required by the crystallizing system to reach a certain value of *X*_*t*_ in a unit crystallization time. Because of that, its value can be treated as a good probe for an assessment of the overall crystallization rate: the lower it is, the faster the crystallization process proceeds. Fig. S15 (SI) presents the Liu and Mo plots acquired for the investigated iPP/CaDMP systems at *X*_*t*_ between 0.1 and 0.9, whereas Fig. 6 and Table S9 (SI) show the calculated values of *a* and *F(T)* kinetic parameters.

Data presented in Fig. 6, clearly proves that a distribution of the CaDMP particles within the iPP matrix reduced the value of *F*(*T*) and this trend intensified with increasing filler content: *F*(*T*)’s estimated for the material containing 1 wt% of CaDMP were decreased by ca. 32–42% compared to the unmodified polymer, depending on the chosen value of *X*_*t*_. Such behavior indicated that the composite systems required lower undercooling (a driving force of crystallization) to reach the same value of *X*_*t*_ compared with neat iPP.

### Wide-angle X-ray scattering (WAXS) measurements

In order to independently confirm the formation of the β-iPP crystalline phase upon the addition of CaDMP to iPP, the X-ray diffraction technique was applied. Fig. S16 (SI) shows experimental WAXS curves obtained for neat iPP and CaDMP, as well as their composites. Neat polymer exhibited only the reflections corresponding to the α-iPP polymorph – those with the highest intensity were located at the 2*θ* angles of 14.1°, 16.9°, 18.7°, 21.2°, or 21.8°, and resulted from X-ray diffractions occurring on the (110), (040), (130), (111) or (041) crystallographic planes, respectively^2^. On the other hand, all the WAXS profiles recorded for the composite materials exhibited an additional, distinct diffraction reflection with maximum around 2*θ* of 16.1°, which could be assigned solely to the β-iPP crystal phase, i.e., the diffraction incidents occurring on its (110) crystallographic plane^2^. Furthermore, the intensity of a diffraction reflection at 2*θ* ca. 21.2° was increased compared to the WAXS trace of neat iPP due to an overlapping of the strong (111) reflections characteristic for both the α and β polymorphs of iPP^2^. The presence of the X-ray signals characteristic for both crystalline forms of iPP confirmed the results of the previously discussed DSC measurements and proved that upon their incorporation into the polymer matrix CaDMP particles exhibited a nucleation ability toward the β-iPP polymorph.

It should be noted, that a careful deconvolution of the experimental WAXS profiles (a detailed description of this procedure can be found in SI, together with its graphical example presented in Fig. S17) allowed us to calculate both the overall crystallinity ($\:{X}_{\text{c}}^{\text{W}\text{A}\text{X}\text{S}}$) and the relative content of the β-iPP crystal phase ($\:{k}_{{\upbeta\:}}^{\text{W}\text{A}\text{X}\text{S}}$). The latter parameter was determined for the two-phase (α-iPP + β-iPP) case, using an empirical equation proposed by Turner Jones and coworkers (Eq. (9))^89^:

9 $$\:{k}_{{\upbeta\:}}^{\text{W}\text{A}\text{X}\text{S}}={I}_{{\upbeta\:}\left(110\right)}^{\text{W}\text{A}\text{X}\text{S}}/\left({I}_{{\upbeta\:}\left(110\right)}^{\text{W}\text{A}\text{X}\text{S}}+{I}_{{\upalpha\:}\left(110\right)}^{\text{W}\text{A}\text{X}\text{S}}+{I}_{{\upalpha\:}\left(040\right)}^{\text{W}\text{A}\text{X}\text{S}}+{I}_{{\upalpha\:}\left(130\right)}^{\text{W}\text{A}\text{X}\text{S}}\right)$$

in which $\:{I}_{{\upbeta\:}\left(110\right)}^{\text{W}\text{A}\text{X}\text{S}}$ is the integral intensity of a diffraction reflection characteristic for the (110) plane of β-iPP, whereas $\:{I}_{{\upalpha\:}\left(110\right)}^{\text{W}\text{A}\text{X}\text{S}}$, $\:{I}_{{\upalpha\:}\left(040\right)}^{\text{W}\text{A}\text{X}\text{S}}$ and $\:{I}_{{\upalpha\:}\left(130\right)}^{\text{W}\text{A}\text{X}\text{S}}$ are the integral intensities of the strongest reflections ascribed to the (110), (040) and (130) planes of the α-iPP crystal phase, respectively. Table 5 presents the results of all above-mentioned calculations.

**Table 5:** Selected parameters evaluated using WAXS analysis of the crystal phases formed within the samples of neat iPP and its composites with cadmp.

CaDMP content/wt%	$\:{I}_{{\upalpha\:}\left(110\right)}^{\text{W}\text{A}\text{X}\text{S}}/{I}_{{\upalpha\:}\left(040\right)}^{\text{W}\text{A}\text{X}\text{S}}$ratio	$\:{X}_{\text{c}}^{\text{W}\text{A}\text{X}\text{S}}$/%	$\:{k}_{{\upbeta\:}}^{\text{W}\text{A}\text{X}\text{S}}$
0.0	1.45	48.8	nd
0.2	0.85	52.6	0.17
0.5	0.78	53.5	0.14
1.0	0.57	53.8	0.36
2.0	0.47	52.6	0.38
5.0	0.48	57.1	0.27

A comparison of the data presented in Tables 3 and 5 led to a conclusion that the processing conditions applied during the preparation of the samples intended for WAXS did not substantially impede their overall crystallization degree since the $\:{X}_{\text{c}}^{\text{W}\text{A}\text{X}\text{S}}$ values were on a comparable level with those estimated from the DSC measurement carried out under non-isothermal conditions: upon an incorporation of the CaDMP particles only a small increase in the amount of the iPP crystalline phase was observed. On the other hand, a distribution of the α- and β-iPP crystallites was drastically changed, since the $\:{k}_{{\upbeta\:}}^{\text{W}\text{A}\text{X}\text{S}}$ values were approximately 2–4 times lower than the $\:{k}_{{\upbeta\:}}^{\text{D}\text{S}\text{C}}$ values determined by a calorimetric method. One can assume, that such a large dominance of the α-iPP polymorph was probably related to a different thermo-mechanical history of the samples subjected to the WAXS and DSC measurements (i.e., different temperature profiles and heat transportation within the samples during their preparation and measurement, as well as a slow β-iPP to α-iPP transformation during cooling and/or subsequent prolonged treatment of the samples at room temperature prior to the WAXS measurement).

It is worth noting, that in the case of the CaDMP-containing materials the intensity of the diffraction peaks related to the (110) and (040) crystallographic planes of α-iPP substantially changed compared to neat iPP: the former decreased its intensity, whereas the latter became a dominant one among the iPP-related reflections (see Fig. S16 (SI)). It suggests that during crystallization in these systems, an orientation of the α-iPP crystallites perpendicular to the (040) plane was favored. As reported in the literature^90^, a ratio of the intensities of these two diffraction peaks ($\:{I}_{{\upalpha\:}\left(110\right)}^{\text{W}\text{A}\text{X}\text{S}}/{I}_{{\upalpha\:}\left(040\right)}^{\text{W}\text{A}\text{X}\text{S}}$) provides information on the extent of the orientation of the α-iPP crystals along its *a*– or *b*–unit cell directions. As can be seen from the data presented in Table 5, the substantially lower values of the $\:{I}_{{\upalpha\:}\left(110\right)}^{\text{W}\text{A}\text{X}\text{S}}/{I}_{{\upalpha\:}\left(040\right)}^{\text{W}\text{A}\text{X}\text{S}}$ ratio estimated for all of the CaDMP-based composites indicated a preferred alignment of the α-iPP crystals in the *b*-axis direction.

### Mechanism of the β-iPP nucleation by CaDMP

**Fig. 7:**
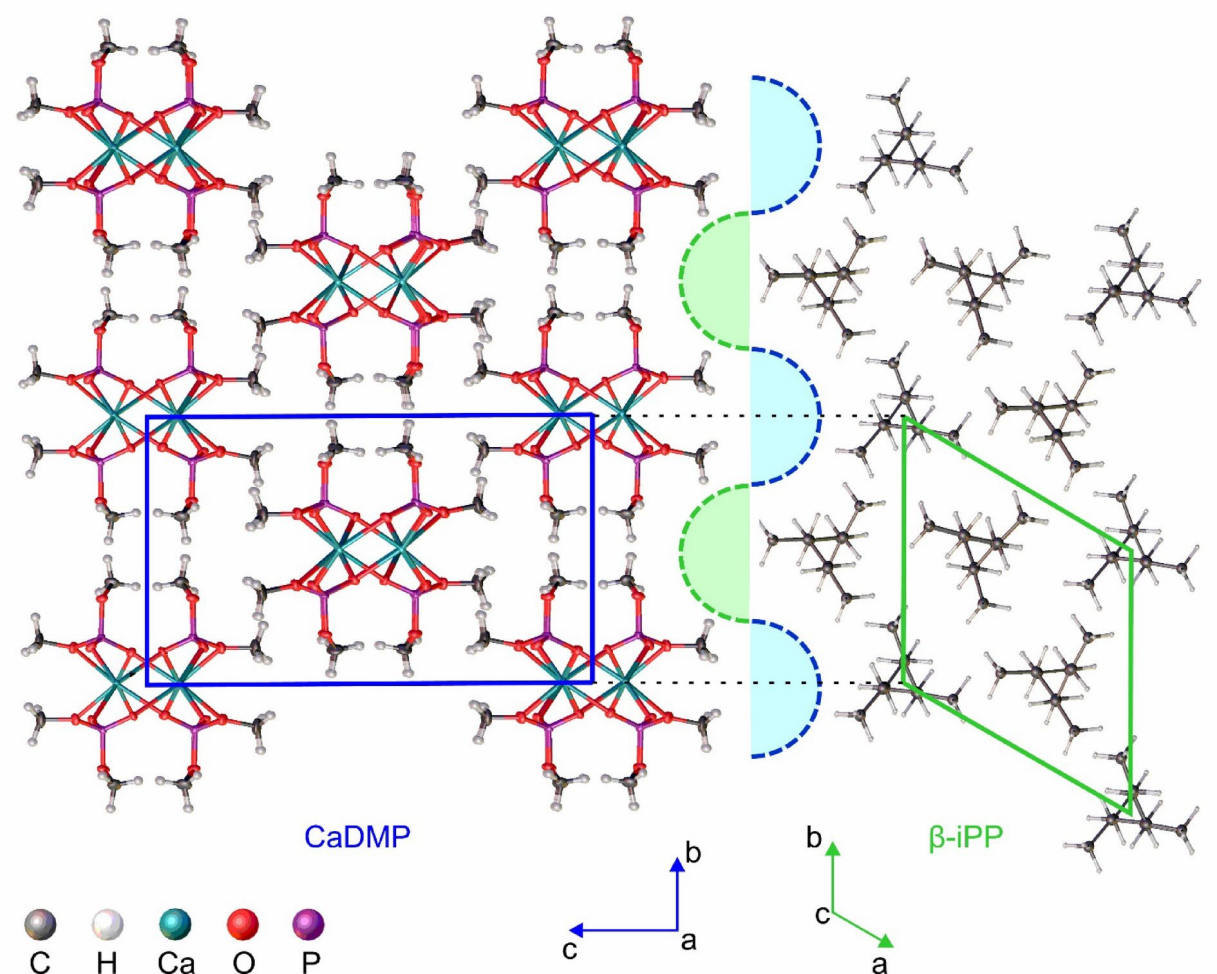
Proposed crystal lattice matching between CaDMP and β-iPP. The data for the β-iPP structure are taken from the work of Meille and coworkers^91^. Unit cell borders and crystallographic directions are indicated with solid lines and arrows, respectively.

The nucleating properties of different compounds towards the formation of β-iPP are usually explained based on a spatial organization and epitaxial growth of the iPP chains at the surface of the NA particles. This process requires the occurrence of an appropriate degree of geometrical matching between the NA lattice parameters and those of the β-iPP crystal phase, which can be quantified with the mismatch coefficient (*f*_m_) defined by Eq. 10:

10 $$\:{f}_{m}=\left[\left({L}_{{\upbeta\:}-\text{i}\text{P}\text{P}}-{L}_{\text{N}\text{A}}\right)/\left({L}_{\text{N}\text{A}}\right)\right]\times\:100\%$$

where *L*_β−iPP_ and *L*_NA_ refer to some specific distance (usually a unit cell parameter or its multiplication) in the crystal structure of β-iPP and NA, respectively^23,24,36,47^. Based on the empirical data, it is assumed that a value of *f*_m_ higher than 15% indicates too much stress between both crystal phases effectively preventing epitaxy^36,47,90^. In the case of β-iPP and CaDMP, a favorable crystallographic matching (*f*_m_ ≈ 0.11% at 30 °C) occurred only between the *b*-axis of the nucleator and *a*- or *b*-axis of the polymer – in both crystal structures these axes are perpendicular to the direction in which the respective polymeric chains (i.e., CaDMP or β-iPP) propagate. Taking this into account, we propose that during the first stage of an epitaxial crystallization iPP macromolecules got oriented parallel to the crystallographic *a*-direction of CaDMP and took position within the gaps formed by the methyl groups from CaDMP chains located at the vertices of the latter’s unit cell. Since the 3_1_ screw axes of these iPP chains laid on the (020) crystallographic plane of CaDMP, the interchain distance specific for the β-iPP crystal phase (11.01 Å) was introduced to the system (Fig. 7). Due to the interactions with this first layer of iPP macromolecules the next layers of polymer chains underwent preorientation and were forced to “reproduce” spatial configuration of the β-iPP phase. It is worth noting, that one-dimensional matching of the iPP interchain distance with the gaps on the *a*–*b* crystallographic plane of CaDMP was crucial for the subsequent growth of the β-iPP phase, since it prevailed over the structural strains caused by an incompatibility between the periodicities of CaDMP and iPP chains (*f*_m_ for the *c*- and *a*-lattice parameters of iPP and CaDMP, respectively, reaches value of ca. 18%). However, one cannot exclude the possibility that at the actual temperatures of the β-iPP crystallization this discrepancy may have got smaller due to the differences in β-iPP and CaDMP thermal expansion coefficients.

### Mechanical properties of the iPP/CaDMP composites

Considering that iPP is a construction material with a broad field of applications, the mechanical properties of neat iPP and its composites with 0.2–5.0 wt% of CaDMP were characterized in tensile and impact tests.

As can be seen in Table 6, the addition of CaDMP to iPP had no statistically significant effect on tensile strength of the obtained materials – only in the case of the system with the highest CaDMP content the observed ca. 5% reduction in tensile strength (in relation to neat iPP) exceeded the limits of measurement error. On the other hand, CaDMP significantly affected elongation at break of iPP, especially at lower ranges of the filler’s concentration: an introduction of only 0.2 wt% of CaDMP changed this parameter from 37% (neat iPP) to 280% (an increase by ca. 657%). The maximum of ductile properties was reached at the 0.5 wt% CaDMP loading, and further increasing the amount of CaDMP in the system resulted in a decrease in its elongation at break: the iPP/5CaDMP composite had it on a comparable level as neat iPP. Moreover, the presence of CaDMP also affected the behavior of the iPP matrix during impact, since all of the obtained iPP/CaDMP composites exhibited increased impact strength compared to the unmodified iPP.

**Table 6:** Selected mechanical parameters of iPP and its composites with CaDMP estimated during tensile and impact tests.

CaDMP content/wt%	Tensile strength/MPa	Elongation at break/%	Impact strength/kJ m
0.0	37.7 ± 0.5	37 ± 8	5.57 ± 0.18
0.2	36.7 ± 0.6	280 ± 56	8.03 ± 0.35
0.5	36.4 ± 0.5	517 ± 51	7.58 ± 0.24
1.0	36.1 ± 2.5	190 ± 71	7.26 ± 0.61
2.0	36.9 ± 0.7	175 ± 7	7.83 ± 0.89
5.0	35.7 ± 0.9	49 ± 3	6.65 ± 0.13

## Conclusion

The results of our studies on CaDMP indicate that its 1D particles consist of infinite polymeric chains built of octahedrally coordinated Ca^2+^ cations bridged by tridentate [(CH_3_O)_2_PO_2_]^−^ ligands. In each dimethylphosphate ligand an oxygen atom of one of its methoxyl groups and oxide ligands from the P=O and dissociated PO-H moieties are involved in a coordination of Ca^2+^ centers. Because of that, CaDMP is free of any auxiliary ligands. This structure is very stable and upon heating up to the beginning of thermal decomposition, it does not undergo any structural transformations apart from a thermal expansion. Under oxidative conditions, CaDMP decomposes above ca. 260–295 °C forming a mixture of inorganic calcium condensed phosphates (i.e., calcium meta- and pyrophosphates).

The incorporation of even a small amount of CaDMP particles results in a substantial increase in both ductility and impact strength of the iPP polymer matrix (e.g., an increase in elongation at break and impact resistance by ca. 657% and 44%, respectively, for iPP/0.2CaDMP), with its tensile strength being practically unchanged. However, taking into account the values of kinetic parameters derived from non-isothermal crystallization experiments (which best reflect the real conditions occurring during processing of the iPP-based materials) and mechanical properties estimated for the injection moulded specimens, one can propose 2 wt% as the optimal level of CaDMP loading among the investigated iPP composites.

DSC and WAXS give evidence for the formation of a mixture of α-iPP and β-iPP crystal domains within the investigated composites. The values of $\:{k}_{\beta\:}^{\text{D}\text{S}\text{C}}$ (0.4–0.6) show that CaDMP exhibits a non-selective, α/β nucleating properties towards iPP. A comparison of the CaDMP and β-iPP unit cell parameters shows an almost perfect one-dimensional matching of their crystal structures in directions perpendicular to the axes of propagation of their chains. However, one cannot exclude the possibility that at the actual temperature of epitaxial crystallization of β-iPP, some additional crystal lattice matching may occur due to the differences in thermal expansion coefficients of β-iPP and CaDMP.

DSC data showed that under isothermal conditions one can observe a substantial increase in the Avrami overall crystallization rate constant of iPP, dependent on the CaDMP content in the system. Similarly, on cooling from the molten state, the evolution of crystallization heat in the iPP/CaDMP composites begins and reaches a maximum rate at temperatures a few Celsius degrees higher than in neat polymer; the resulting crystallization exotherms also become narrower than the reference one. One can conclude that the iPP non-isothermal crystallization kinetics are also positively affected by CaDMP particles. Mathematical modelling of this process following the Liu-Mo theory reveals that, with increasing CaDMP content, the composite systems requires lower supercooling to reach the same relative degree of crystallinity in a unit time, as showed by lower values of the Liu-Mo cooling function, *F*(*T*).

## Electronic supplementary material

Below is the link to the electronic supplementary material.

Supplementary Material 1

## Data Availability

The authors declare that the data supporting the findings of this study are included in this published article (and its Supplementary Information files). Should any raw data files be needed, they are available from the corresponding author upon reasonable request.Crystallographic data for the structure reported in this paper have been deposited in the Cambridge Crystallographic Data Centre with CCDC number 2173285. These data can be obtained free of charge via www.ccdc.cam.ac.uk/data_request/cif.
