# Supplementary material for: Calcium coordination polymer containing dimethylphosphate ligands and exhibiting nucleating properties towards α and Β crystal polymorphs of isotactic polypropylene
Source: Sci Rep. 2025 May 2;15:15447. doi: 10.1038/s41598-025-99757-4 (PMC12048558; doi:10.1038/s41598-025-99757-4)
Supplement: Supplementary file 1 — Supplementary Material 1 [file 41598_2025_99757_MOESM1_ESM.pdf]

Scientific Reports

Supporting Information (SI) for

Calcium coordination polymer containing dimethylphosphate ligands and exhibiting nucleating properties towards α and β crystal polymorphs of isotactic polypropylene

Maciej Dębowski[a]*, Mateusz Kullas[b], Krystyna Czaja[b] Beata Sacher-Majewska[b], Marcin Bączek[c], Maciej Dranka[a], Andrzej Ostrowski[a], Zbigniew Florjańczyk[a]

[a]*Faculty of* Chemistry*, Warsaw University of Technology, Noakowskiego 3, 00-664 Warsaw, Poland*

[b]*Institute of Chemistry, University of Opole, Oleska 48, 45-052 Opole, Poland*

[c]*Faculty of Materials, Civil and Environmental Engineering, University of Bielsko-Biała, Willowa 2, 43-309 Bielsko-Biała, Poland*

*Corresponding author. maciej.debowski@pw.edu.pl

TABLE OF CONTENTS

	Short Description	Page
Table S1	Results of elemental analysis of CaDMP	S3
Table S2	Details of a single-crystal structure analysis of CaDMP	S3
Table S3	Selected atom distances (A…B) and bond lengths (A–B) for CaDMP	S4
Table S4	Selected angles between atoms in CaDMP crystal structure	S4
Table S5	Thermogravimetric data for CaDMP measured in air	S5
Table S6	BVV calculations for calcium and phosphorus centers within the CaDMP structure	S6
Table S7	Lattice parameters and cell volume calculated from the VT–PXRD patterns collected at the selected temperatures (27–235 °C)	S7
Table S8	Coefficients of the volumetric and linear thermal expansion of CaDMP crystal phase	S8
Table S9	Liu and Mo's kinetics parameters determined during a non-isothermal crystallization of iPP and its composites with 0.2 or 1.0 wt% of CaDMP	S8
Fig. S1	FTIR spectrum of CaDMP	S9
Fig. S2	^{31}P solid state MAS NMR spectrum of CaDMP	S9
Fig. S3	^{31}P NMR spectrum of CaDMP recorded in CD_3OD	S9
Fig. S4	^{1}H NMR spectrum of CaDMP recorded in CD_3OD	S10
Fig. S5	Thermogravimetric analysis of CaDMP carried out in air	S10
Fig. S6	PXRD trace of a solid residue from CaDMP thermolysis carried out in air, with the reflexes characteristic of $Ca(PO_3)_2$ and $Ca_2P_2O_7$	S11
Fig. S7	Thermogravimetric analysis of CaDMP coupled with a quadrupole mass spectrometry (QMS) of the evolved gases	S12
Fig. S8	Crystal structure of CaDMP: asymmetric unit and coordination sphere of calcium cat-ion	S13
Fig. S9	Packing of CaDMP chains in a view along the crystallographic *x*-axis	S14
Fig. S10	SEM images of CaDMP particles	S15
Fig. S11	DSC trace of CaDMP recorded between −150 and 220 °C	S16
Fig. S12	The effect of temperature on the CaDMP lattice parameters and unit cell volume	S16
Fig. S13	Analysis of the β-iPP fraction based on the DSC melting profiles	S17
Fig. S14	Non-isothermal crystallization of neat iPP and its composites with 0.2 wt% or 1.0 wt% of CaDMP. DSC exotherms recorded at different cooling rates and the variation of a relative crystallinity with crystallization time	S18
Fig. S15	Liu and Mo plots for iPP and its composites filled with 0.2 wt% or 1.0 wt% of CaDMP	S19
Fig. S16	X-ray diffraction reflections of isotactic polypropylene from a deconvolution of the experimental WAXS curves recorded for neat iPP and its composite with 1 wt% of CaDMP.	S20
	Supporting Information references	S21

*Corresponding author. maciej.debowski@pw.edu.pl

SUPPORTING INFORMATION TABLES

Table S1 Elemental analysis of CaDMP

CaDMP formula		Results of elemental analysis		
		Carbon/wt%	Hydrogen/wt%	Calcium/wt%
$C_4H_{12}CaO_8P_2$	Theoretical values [a]	16.56	4.17	13.81
	Experimental values	16.49	4.26	13.2

[a] values calculated for the assumed CaDMP formula

Table S2 Crystal data and structure refinement details for CaDMP

Compound	CaDMP
Empirical formula	$C_4H_{12}CaO_8P_2$
Formula mass	290.16
Temperature/K	120.0(1)
Crystal system	monoclinic
Space group	$P2_1/c$
a/Å	5.4705(2)
b/Å	10.8408(4)
c/Å	18.0457(7)
β/°	90.870(3)
Volume/Å^3	1,070.07(7)
Z	4
ρ_{calcd}/kg m^{-3}	1,801
μ/mm^{-1}	0.906
F(000)	600.0
Crystal size/mm^3	0.15×0.15×0.5
Radiation	MoKα (λ = 0.7107)
Reflections collected	5,008
Independent reflections	5,008
Restraints/parameters	0/141
Goodness-of-fit on F^2	1.068
Final R_1 ($I > 2\sigma(I)$)	0.0283
Final w$R(F^2)$ ($I > 2\sigma(I)$)	0.0758
Final R_1 (all data)	0.0329
Final w$R(F^2)$ (all data)	0.0773
Residual density: max/min/e Å^{-3}	0.43/−0.41
CCDC number	2173285

*Corresponding author. maciej.debowski@pw.edu.pl

Table S3 Selected atom distances (A…B) and bond lengths (A–B) for CaDMP

Atom A	Atom B	Length/Distance/Å	Atom A	Atom B	Length/Distance/Å
Ca(1)	Ca(1)^{a}	3.7304(7)	P(1)	O(3)	1.6055(14)
Ca(1)	O(1)	2.3603(13)	P(1)	O(4)	1.5950(15)
Ca(1)	O(2)^{b}	2.4165(12)	P(2)	O(11)	1.4826(12)
Ca(1)	O(2)^{c}	2.4252(14)	P(2)	O(12)	1.5001(14)
Ca(1)	O(3)^{b}	2.6770(14)	P(2)	O(13)	1.6016(14)
Ca(1)	O(11)	2.3618(13)	P(2)	O(14)	1.5907(15)
Ca(1)	O(12)^{a}	2.4271(14)	O(3)	C(1)	1.433(3)
Ca(1)	O(12)^{d}	2.4085(13)	O(4)	C(2)	1.445(2)
Ca(1)	O(13)^{d}	2.6920(14)	O(13)	C(11)	1.438(3)
P(1)	O(1)	1.4804(12)	O(14)	C(12)	1.446(2)
P(1)	O(2)	1.5018(13)			

Symmetry codes: ^{a} $-x$,1$-y$,1$-z$; ^{b} $-1+x$,$+y$,$+z$; ^{c} 1$-x$,1$-y$,1$-z$; ^{d} 1$+x$,$+y$,$+z$;

Table S4 Selected angles between atoms in CaDMP crystal structure

Atom A	Atom B	Atom C	ABC angle/°	Atom A	Atom B	Atom C	ABC angle/°
O(1)	Ca(1)	Ca(1)^{a}	139.59(4)	O(1)	P(1)	Ca(1)^{d}	129.90(6)
O(1)	Ca(1)	O(2)^{b}	107.13(5)	O(1)	P(1)	O(2)	120.85(8)
O(1)	Ca(1)	O(2)^{c}	151.37(5)	O(1)	P(1)	O(3)	111.80(8)
O(1)	Ca(1)	O(3)^{b}	75.66(4)	O(1)	P(1)	O(4)	106.72(7)
O(1)	Ca(1)	O(11)	129.70(4)	O(2)	P(1)	Ca(1)^{d}	45.40(5)
O(1)	Ca(1)	O(12)^{d}	84.94(4)	O(2)	P(1)	O(3)	101.06(7)
O(1)	Ca(1)	O(12)^{a}	77.91(5)	O(2)	P(1)	O(4)	110.65(8)
O(1)	Ca(1)	O(13)^{d}	73.51(5)	O(3)	P(1)	Ca(1)^{d}	55.97(5)
O(2)^{b}	Ca(1)	Ca(1)^{a}	39.69(3)	O(4)	P(1)	Ca(1)^{d}	123.31(5)
O(2)^{c}	Ca(1)	Ca(1)^{a}	39.52(3)	O(4)	P(1)	O(3)	104.66(7)
O(2)^{b}	Ca(1)	O(2)^{c}	79.21(5)	O(11)	P(2)	Ca(1)^{b}	130.41(6)
O(2)^{c}	Ca(1)	O(3)^{b}	126.97(4)	O(11)	P(2)	O(12)	120.58(8)
O(2)^{b}	Ca(1)	O(3)^{b}	55.93(4)	O(11)	P(2)	O(13)	112.05(8)
O(2)^{b}	Ca(1)	O(12)^{a}	76.55(4)	O(11)	P(2)	O(14)	106.60(7)
O(2)^{c}	Ca(1)	O(12)^{a}	76.56(4)	O(12)	P(2)	Ca(1)^{b}	44.86(5)
O(2)^{c}	Ca(1)	O(13)^{d}	112.96(5)	O(12)	P(2)	O(13)	100.95(7)
O(2)^{b}	Ca(1)	O(13)^{d}	154.33(4)	O(12)	P(2)	O(14)	111.08(8)
O(3)^{b}	Ca(1)	Ca(1)^{a}	91.61(3)	O(13)	P(2)	Ca(1)^{b}	56.32(5)
O(3)^{b}	Ca(1)	O(13)^{d}	100.99(4)	O(14)	P(2)	Ca(1)^{b}	122.95(5)

*Corresponding author. maciej.debowski@pw.edu.pl

Table S4 Selected angles between atoms in CaDMP crystal structure (*continued)*

Atom A	Atom B	Atom C	ABC angle/°	Atom A	Atom B	Atom C	ABC angle/°
O(11)	Ca(1)	Ca(1)^{a}	79.71(4)	O(14)	P(2)	O(13)	104.50(7)
O(11)	Ca(1)	O(2)^{c}	77.79(5)	P(1)	O(1)	Ca(1)	130.67(9)
O(11)	Ca(1)	O(2)^{b}	86.37(4)	Ca(1)$^{4)}$	O(2)	Ca(1)^{c}	100.79(5)
O(11)	Ca(1)	O(3)^{b}	73.05(5)	P(1)	O(2)	Ca(1)^{d}	108.33(7)
O(11)	Ca(1)	O(12)^{a}	151.37(5)	P(1)	O(2)	Ca(1)^{c}	135.29(7)
O(11)	Ca(1)	O(12)^{d}	107.82(5)	P(1)	O(3)	Ca(1)^{d}	94.23(7)
O(11)	Ca(1)	O(13)^{d}	75.02(4)	C(1)	O(3)	Ca(1)^{d}	137.95(12)
O(12)^{d}	Ca(1)	Ca(1)^{a}	114.69(4)	C(1)	O(3)	P(1)	121.40(12)
O(12)^{a}	Ca(1)	Ca(1)^{a}	72.44(3)	C(2)	O(4)	P(1)	117.95(12)
O(12)^{d}	Ca(1)	O(2)^{b}	149.35(4)	P(2)	O(11)	Ca(1)	129.56(9)
O(12)^{d}	Ca(1)	O(2)^{c}	77.60(4)	Ca(1)^{b}	O(12)	Ca(1)^{a}	101.24(5)
O(12)^{a}	Ca(1)	O(3)^{b}	113.60(5)	P(2)	O(12)	Ca(1)^{b}	109.08(7)
O(12)^{d}	Ca(1)	O(3)^{b}	153.59(4)	P(2)	O(12)	Ca(1)^{a}	136.31(7)
O(12)^{d}	Ca(1)	O(12)^{a}	78.76(5)	P(2)	O(13)	Ca(1)^{b}	94.00(7)
O(12)^{d}	Ca(1)	O(13)^{d}	55.63(4)	C(11)	O(13)	Ca(1)^{b}	143.89(12)
O(12)^{a}	Ca(1)	O(13)^{d}	127.27(4)	C(11)	O(13)	P(2)	120.83(12)
O(13)^{d}	Ca(1)	Ca(1)^{a}	146.88(4)	C(12)	O(14)	P(2)	118.16(12)

Symmetry codes: ^{a} $-x,1-y,1-z$; ^{b} $-1+x,+y,+z$; ^{c} $1-x,1-y,1-z$; ^{d} $1+x,+y,+z$

Table S5 Thermogravimetric data for CaDMP measured in air

Type of process	T_{b}/°C [a]	$T_{98\%}$/°C [b]	$T_{95\%}$/°C [b]	T_{onset}/°C [c]	T_{endset}/°C [c]	m_{600}/wt% [d]
multistep	257.7	279.4	285.9	275.7	378.1	52.8

[a] temperature of the beginning of a mass loss (a bend point on the TG curve); [b] $T_{98\%}$ and $T_{95\%}$ denote temperatures at which sample mass reached 98% and 95% of its initial value, respectively; [c] T_{onset} and T_{endset} denote the extrapolated onset and endset temperatures of a mass loss, respectively; [d] residual mass of the sample measured at 600 °C (value expressed as % of the initial sample mass)

*Corresponding author. maciej.debowski@pw.edu.pl

Calculations of bond–valence vectors for calcium and phosphorus centers in CaDMP

Bond valences were calculated using the most widely used equation describing the relationship between the bond length (d_{ij}) between the *i*–th and *j*–th atoms and the valence of this bond (s_{ij}), Eq. (S1) [S1]:

$$s_{ij} = \exp[(r_{ij} - d_{ij}) \times b^{-1}] \quad \text{(S1)}$$

where r_{ij} and b are empirically determined constants for the given *i*–*j* bond. r_{ij} is equal to the length of a conceptual bond of a unit valence, whereas the parameter b is generally treated as a ‘universal’ constant, often taken to be 0.37 Å [S1,S2]. The following parameters were used in the calculations: r_{PO} = 1.615 Å (b = 0.38 Å) and r_{CaO} = 1.927 Å (b = 0.39 Å) [S3], together with the experimental d_{ij} values derived from a single-crystal X-ray analysis of CaDMP.

According to the bond–valence vector model, the bond between the coordination center *i* and the more electronegative ligating atom *j* of s_{ij} valence can be represented by the bond–valence vector $\boldsymbol{v}_{ij}$ directed from *i* to *j* with a length defined by Eq. (S2) [S4]:

$$|\boldsymbol{v}_{ij}| = s_{ij} \times [1 - (s_{ij} \times Q_i^{-1})] \quad \text{(S2)}$$

where Q_i is the charge in the core of the central *i*–th atom. The lengths of the individual bond–valence vectors ($|\boldsymbol{v}_{CaO}|$ and $|\boldsymbol{v}_{PO}|$) were calculated by setting the calcium core charge Q_{Ca} = 2 and the phosphorus core charge Q_P = 5.

The bond–valence sums (S_i) and lengths of the resultant bond–valence vectors ($|\boldsymbol{v}_i|$) for each coordination centers *i* connected to the ligating oxygen atoms *j* were calculated according to the following equations:

$$S_i = \sum_j s_{ij} \quad \text{(S3)}$$

$$\boldsymbol{v}_i = \sum_j \boldsymbol{v}_{ij} \quad \text{(S4)}$$

$$|\boldsymbol{v}_i| = (\boldsymbol{v}_i \cdot \boldsymbol{v}_i)^{0.5} \quad \text{(S5)}$$

Table S6 Bond–valence sum (S_i) and length of the resultant bond–valence vector $|\boldsymbol{v}_i|$ for each crystallographically distinct calcium or phosphorus coordination center in CaDMP

Atom *i*	S_i	$|\boldsymbol{v}_i|$/v.u. [a]
Ca	2.08	0.104
P1	4.85	0.040
P2	4.87	0.044

[a] valence units.

*Corresponding author. maciej.debowski@pw.edu.pl

Calculations of CaDMP unit cell parameters at different temperatures

Table S7 Lattice parameters (a, b, c and β angle) and cell volume (V_{CaDMP}) calculated from the VT-PXRD patterns of CaDMP collected at the selected temperatures

T/°C	a/Å	b/Å	c/Å	β/°	V_{CaDMP}/Å^3	a/Å	b/Å	c/Å	β/°	V_{CaDMP}/Å^3
	VT-PXRD heating mode [a]					*VT-PXRD cooling mode* [b]				
30	5.4804	11.0172	18.1076	89.910	1,093.32	5.4805	11.0168	18.1105	89.905	1,093.47
50	5.4803	11.0393	18.1168	89.930	1,096.04	5.4805	11.0418	18.1202	89.933	1,096.53
70	5.4811	11.0625	18.1259	89.919	1,099.04	5.4815	11.0647	18.1304	89.918	1,099.62
90	5.4824	11.0875	18.1337	90.045	1,102.28	5.4824	11.0875	18.1337	90.045	1,102.28
110	5.4840	11.1120	18.1405	90.055	1,105.44	5.4834	11.1126	18.1429	90.052	1,105.53
120	5.4842	11.1230	18.1426	90.055	1,106.70	5.4837	11.1229	18.1446	90.066	1,106.73
130	5.4833	11.1354	18.1486	90.029	1,108.12	5.4840	11.1385	18.1506	90.095	1,108.70
150	5.4833	11.1609	18.1577	90.029	1,111.23	nd [c]	nd [c]	nd [c]	nd [c]	nd [c]
170	5.4845	11.1900	18.1702	90.098	1,115.12	nd [c]	nd [c]	nd [c]	nd [c]	nd [c]
190	5.4854	11.2205	18.1820	90.139	1,119.08	nd [c]	nd [c]	nd [c]	nd [c]	nd [c]

[a] heating from 30 to 190 °C; [b] cooling from 190 to 30 °C; [c] values excluded from analysis due to insufficient quality of the recorded PXRD traces

A fitting of the experimental data points presented in Table S7 by the least–squares method gives the following equations (in which T is expressed in K) for the a, b, c lattice parameters and the volume of CaDMP unit cell (V_{CaDMP}) – for the cooling mode, data points at 170 and 190 °C were excluded from the analysis:

VT-PXRD heating mode

$a = 3.20(45) \times 10^{-5}\ [\text{Å K}^{-1}]\ T + 5.4706(17)\ [\text{Å}]$ (R^2= 0.8652) (S6)

$b = 12.57(22) \times 10^{-4}\ [\text{Å K}^{-1}]\ T + 10.6319(86)\ [\text{Å}]$ (R^2= 0.9975) (S7)

$c = 4.45(17) \times 10^{-4}\ [\text{Å K}^{-1}]\ T + 17.9715(66)\ [\text{Å}]$ (R^2= 0.9886) (S8)

$V_{\text{CaDMP}} = 15.86(32) \times 10^{-2}\ [\text{Å}^3\ \text{K}^{-1}]\ T + 1{,}044.7(12)\ [\text{Å}^3]$ (R^2= 0.9969) (S9)

VT-PXRD cooling mode

$a = 3.91(30) \times 10^{-5}\ [\text{Å K}^{-1}]\ T + 5.4682(11)\ [\text{Å}]$ (R^2= 0.9719) (S10)

$b = 11.96(15) \times 10^{-4}\ [\text{Å K}^{-1}]\ T + 10.6544(53)\ [\text{Å}]$ (R^2= 0.9992) (S11)

$c = 3.79(20) \times 10^{-4}\ [\text{Å K}^{-1}]\ T + 17.9973(73)\ [\text{Å}]$ (R^2= 0.9859) (S12)

$V_{\text{CaDMP}} = 14.98(20) \times 10^{-2}\ [\text{Å}^3\ \text{K}^{-1}]\ T + 1{,}048.1(7)\ [\text{Å}^3]$ (R^2= 0.9991) (S13)

*Corresponding author. maciej.debowski@pw.edu.pl

Table S8 Coefficients of the volumetric and linear thermal expansion of CaDMP crystal structure [a]

VT-PXRD mode	α_a/K^{-1}	α_b/K^{-1}	α_c/K^{-1}	β_V/K^{-1}	T range/K [b]
heating	5.84(82) ×10^{-6}	11.41(20) ×10^{-5}	2.46(9) ×10^{-5}	14.51(29) ×10^{-5}	303.15–463.15
cooling	7.14(54) ×10^{-6}	10.86(13) ×10^{-5}	2.09(11) ×10^{-5}	13.70(19) ×10^{-5}	303.15–403.15

[a] values estimated according to the previously described method [S5], on the basis of the slopes of the straight lines described by Eqs. (S6)–(S13); [b] temperature range over which the respective thermal expansion coefficients were estimated.

Table S9 Liu and Mo kinetics parameters [a] determined during a non-isothermal crystallization of iPP and its composites with 0.2 or 1.0 wt% of CaDMP

	neat iPP			iPP/0.2CaDMP			iPP/1CaDMP		
X_t	*a*	*F(T)*	R^2 [b]	*a*	*F(T)*	R^2 [b]	*a*	*F(T)*	R^2 [b]
0.1	1.03(18)	8.23(80)	0.9443	1.13(21)	6.53(53)	0.9402	0.968(21)	4.977(50)	0.9991
0.2	1.01(20)	9.6(13)	0.9313	1.13(18)	7.59(61)	0.9551	0.987(23)	5.619(57)	0.9990
0.3	1.00(22)	10.5(17)	0.9145	1.14(16)	8.35(69)	0.9619	1.008(28)	6.122(76)	0.9985
0.4	1.00(24)	11.3(22)	0.8990	1.16(16)	9.03(76)	0.9662	1.031(35)	6.61(11)	0.9977
0.5	1.00(26)	12.0(26)	0.8877	1.17(15)	9.71(85)	0.9692	1.056(43)	7.12(15)	0.9967
0.6	1.01(27)	12.7(30)	0.8805	1.19(15)	10.44(95)	0.9716	1.083(53)	7.70(20)	0.9953
0.7	1.02(27)	13.5(34)	0.8767	1.21(14)	11.3(11))	0.9737	1.113(64)	8.40(29)	0.9934
0.8	1.03(28)	14.6(40)	0.8757	1.24(14)	12.4(13)	0.9757	1.152(79)	9.37(43)	0.9908
0.9	1.06(29)	16.2(48)	0.8772	1.29(14)	14.5(16)	0.9775	1.21(10)	11.11(74)	0.9866

[a] standard deviation of its value is showed in parenthesis; [b] coefficient of determination

*Corresponding author. maciej.debowski@pw.edu.pl

SUPPORTING INFORMATION FIGURES

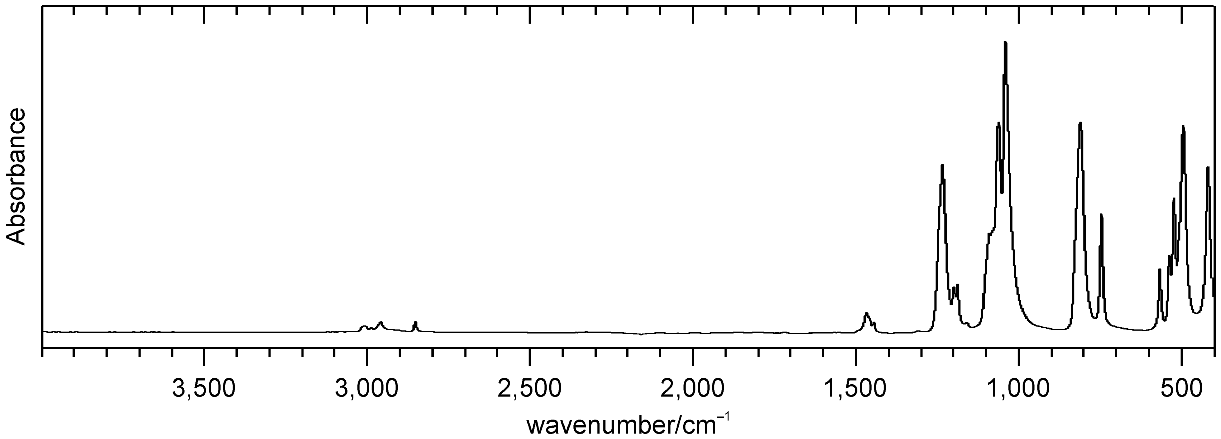

Fig. S1 FTIR spectrum of CaDMP

FTIR (neat sample, ATR-diamond, cm^{-1}): 3,003 (vw), 2,982 (vw), 2,954 (vw), 2,848 (vw), 1,463 (vw), 1,441 (vw), 1,230 (s), 1,194 (w), 1,183 (w), 1,154 (vw), 1,088 (s), 1,060 (vs), 1,037 (vs), 806 (vs), 743 (s), 562 (s), 533 (s), 519 (vs), 493 (vs), 415 (vs)

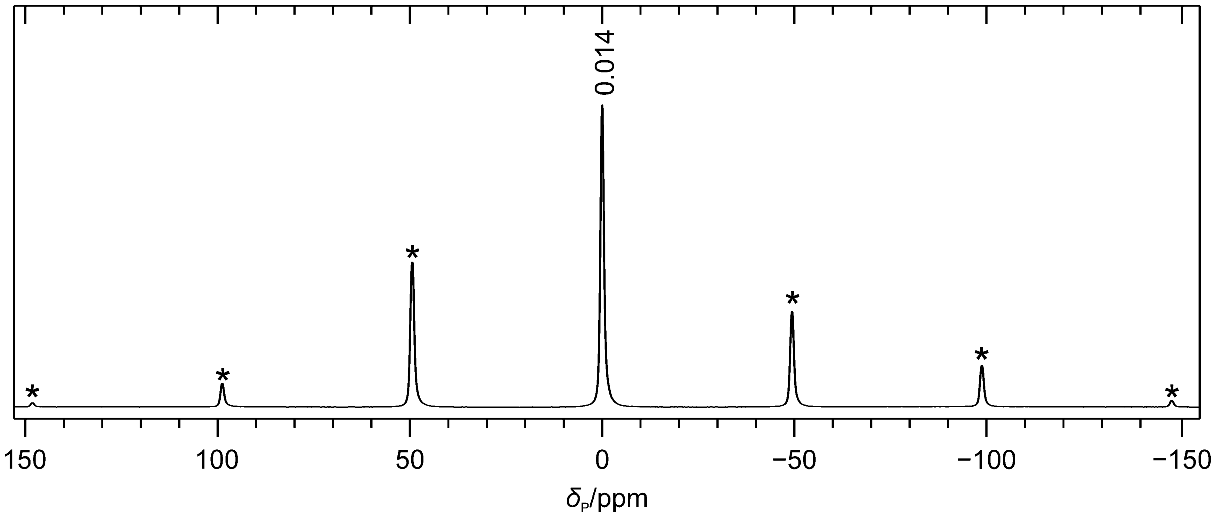

Fig. S2 ^{31}P solid state MAS NMR spectrum of CaDMP. The sidebands are marked with asterisks

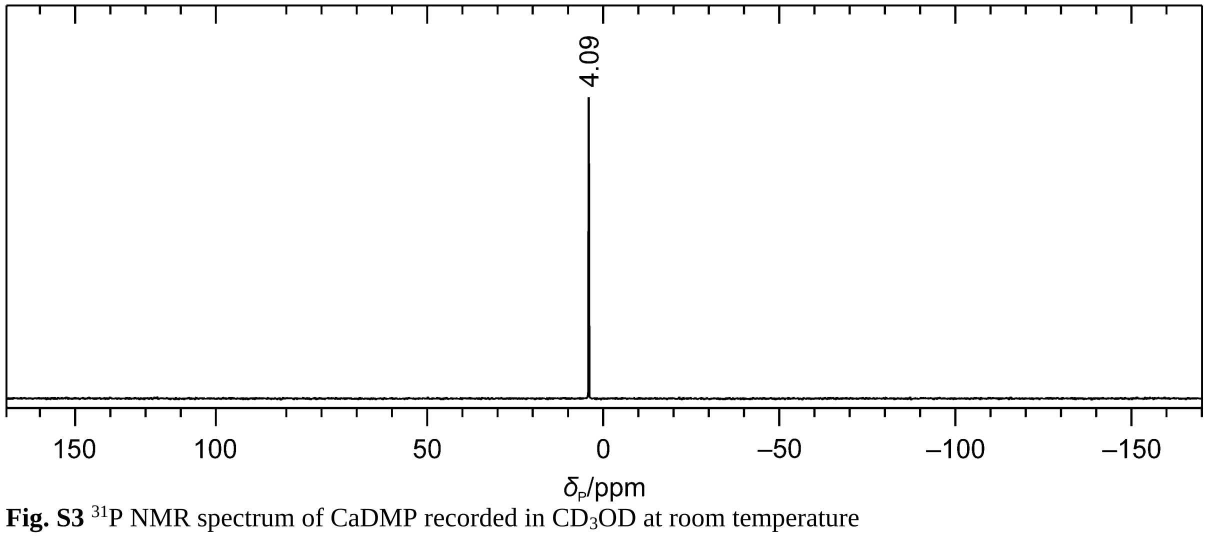

Fig. S3 ^{31}P NMR spectrum of CaDMP recorded in CD_3OD at room temperature

*Corresponding author. maciej.debowski@pw.edu.pl

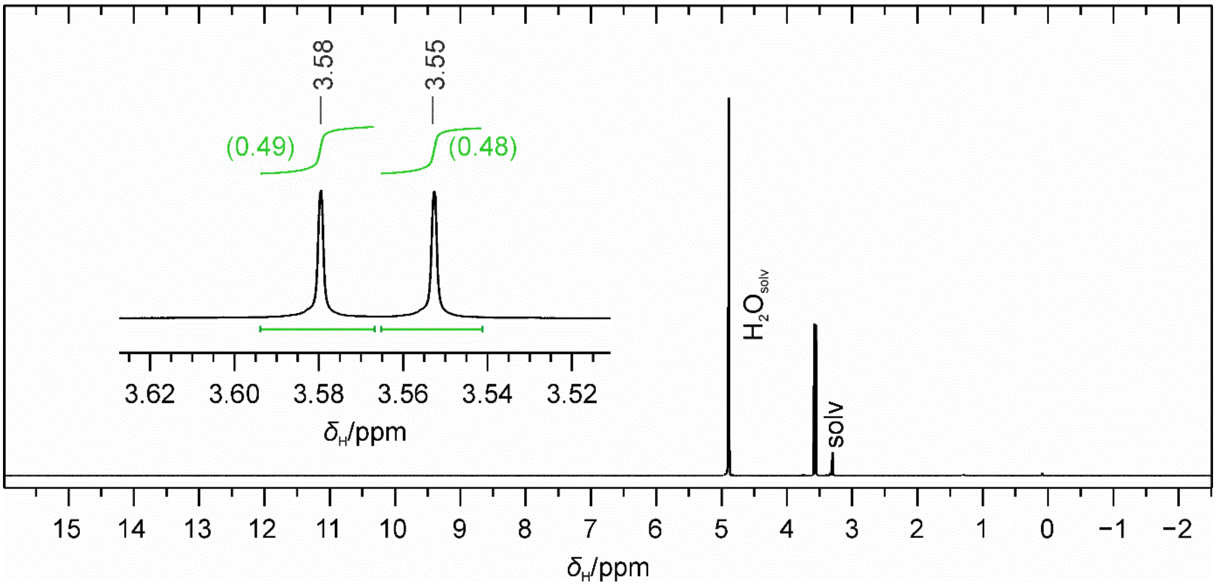

Fig. S4 ^{1}H NMR spectrum of CaDMP recorded in CD_3OD. Abbreviations: solv – solvent residual peak, H_2O_{solv} – water present in solvent. The integrals are presented in parentheses

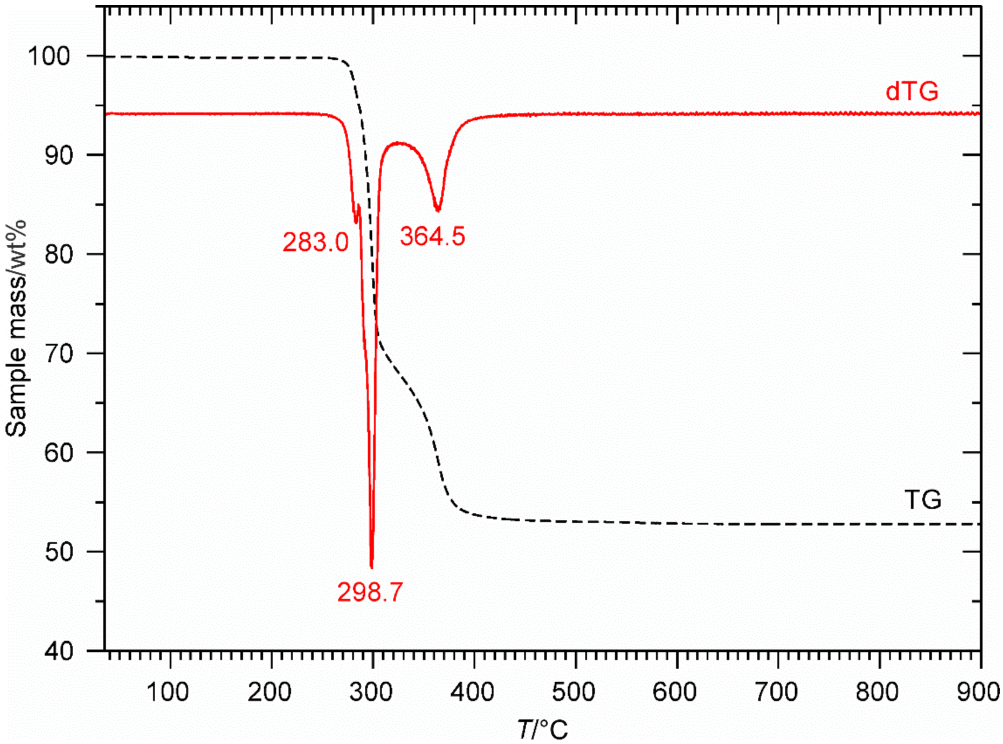

Fig. S5 Thermogravimetric analysis of CaDMP carried out in a stream of synthetic air: TG (black, dashed line) and dTG (red, solid line) curves

*Corresponding author. maciej.debowski@pw.edu.pl

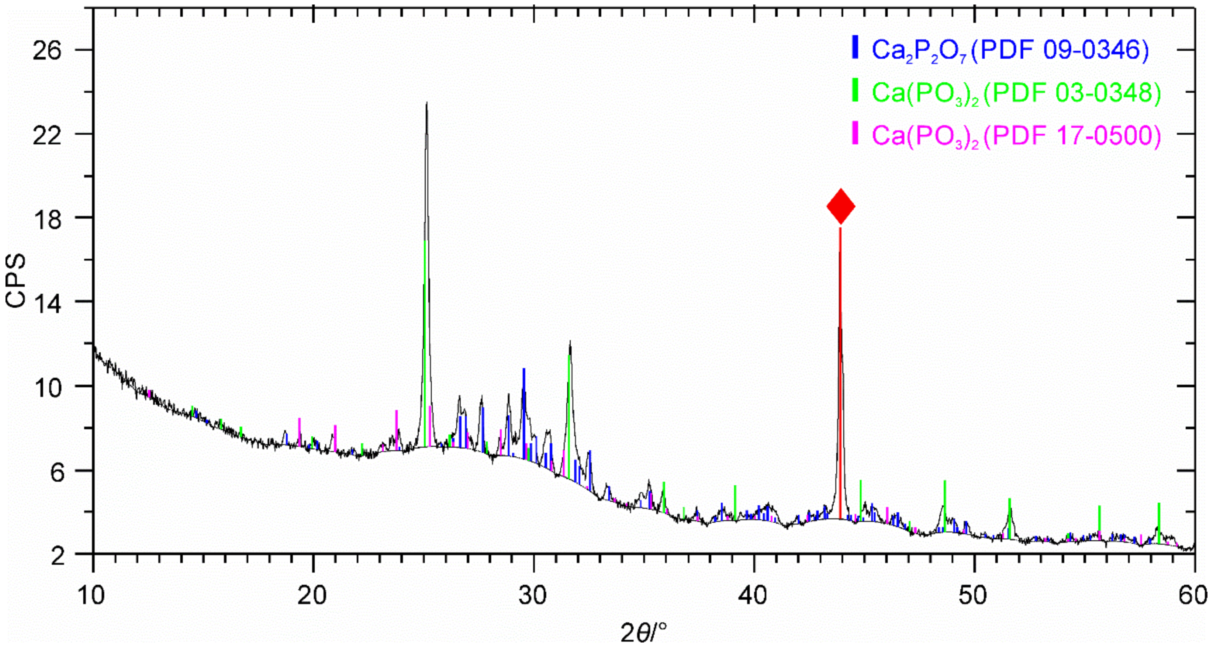

Fig. S6 PXRD trace of a solid residue from CaDMP thermolysis carried out for 6 hours at 600 °C in a stream of air (black line). A red bar and ♦ symbol at 2θ angle of ca. 44° indicate a reflection from diamond powder used as an internal standard. Reflections characteristic of crystal phases of some selected calcium condensed phosphates are indicated with the coloured bars, together with their respective Powder Diffraction File numbers (in square brackets)

*Corresponding author. maciej.debowski@pw.edu.pl

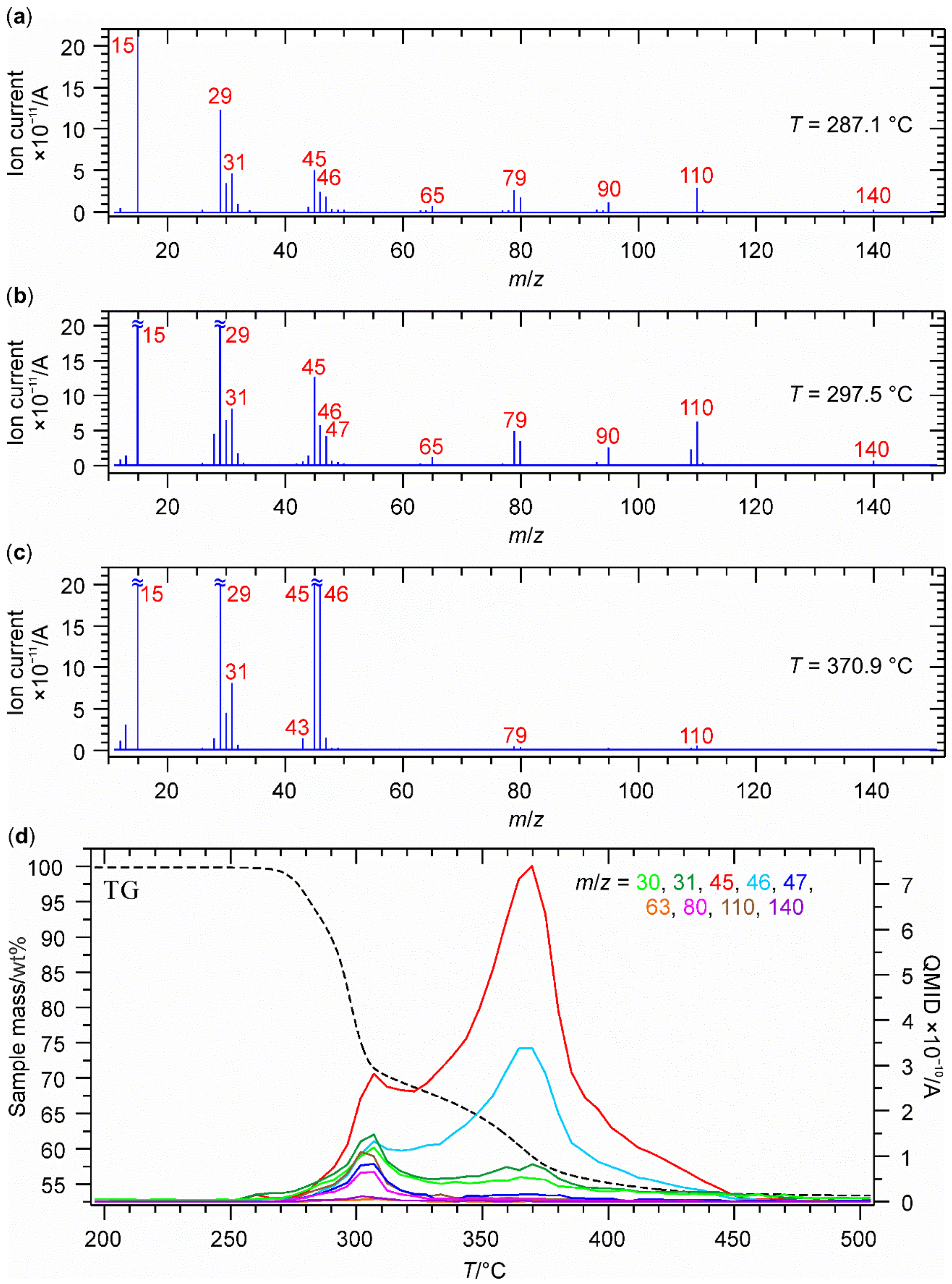

Fig. S7 (**a**)–(**c**) QMS spectra measured at the selected temperatures for the volatiles evolved during STA of CaDMP; (**d**) TG curve (black, dashed line) and QMS curves (coloured, solid lines) for the ions characteristic for: formaldehyde (m/z = 30), methanol (m/z = 31), dimethyl ether (m/z = 45 and 46), as well as dimethylphosphite and TMP molecular ions (m/z = 110 and 140, respectively) and oxophosphorus species: PO (m/z = 47), PO_2 (m/z = 63) and $HOPO_2$ (m/z = 80). For the clarity of presentation the temperature range is limited to the 200–500 °C region

*Corresponding author. maciej.debowski@pw.edu.pl

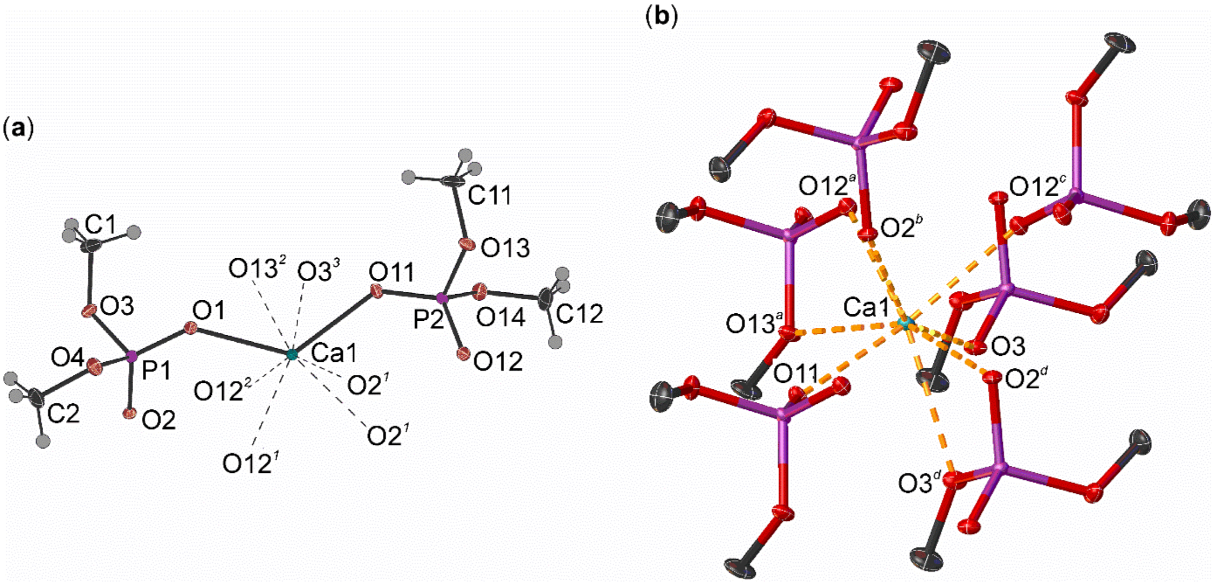

Fig. S8 Crystal structure of CaDMP: (**a**) asymmetric unit showing the atomic numbering scheme and 50% displacement ellipsoids (symmetry codes: [1] 1−*x*,1−*y*,1−*z*; [2] −1+*x*,+*y*,+*z*; [3] −*x*,1−*y*,1−*z*; [4] 1+*x*,+*y*,+*z*), (**b**) fragment showing coordination sphere of calcium cation (symmetry codes: [a] 1+*x*,+*y*,+*z*; [b] 1−*x*,1−*y*,1−*z*; [c] −*x*,1−*y*,1−*z*; [d] −1+*x*,+*y*,+*z*). Dashed lines represent directions of coordinated oxygen atoms from the adjacent DMP molecules. Hydrogen atoms are omitted for the clarity of presentation

*Corresponding author. maciej.debowski@pw.edu.pl

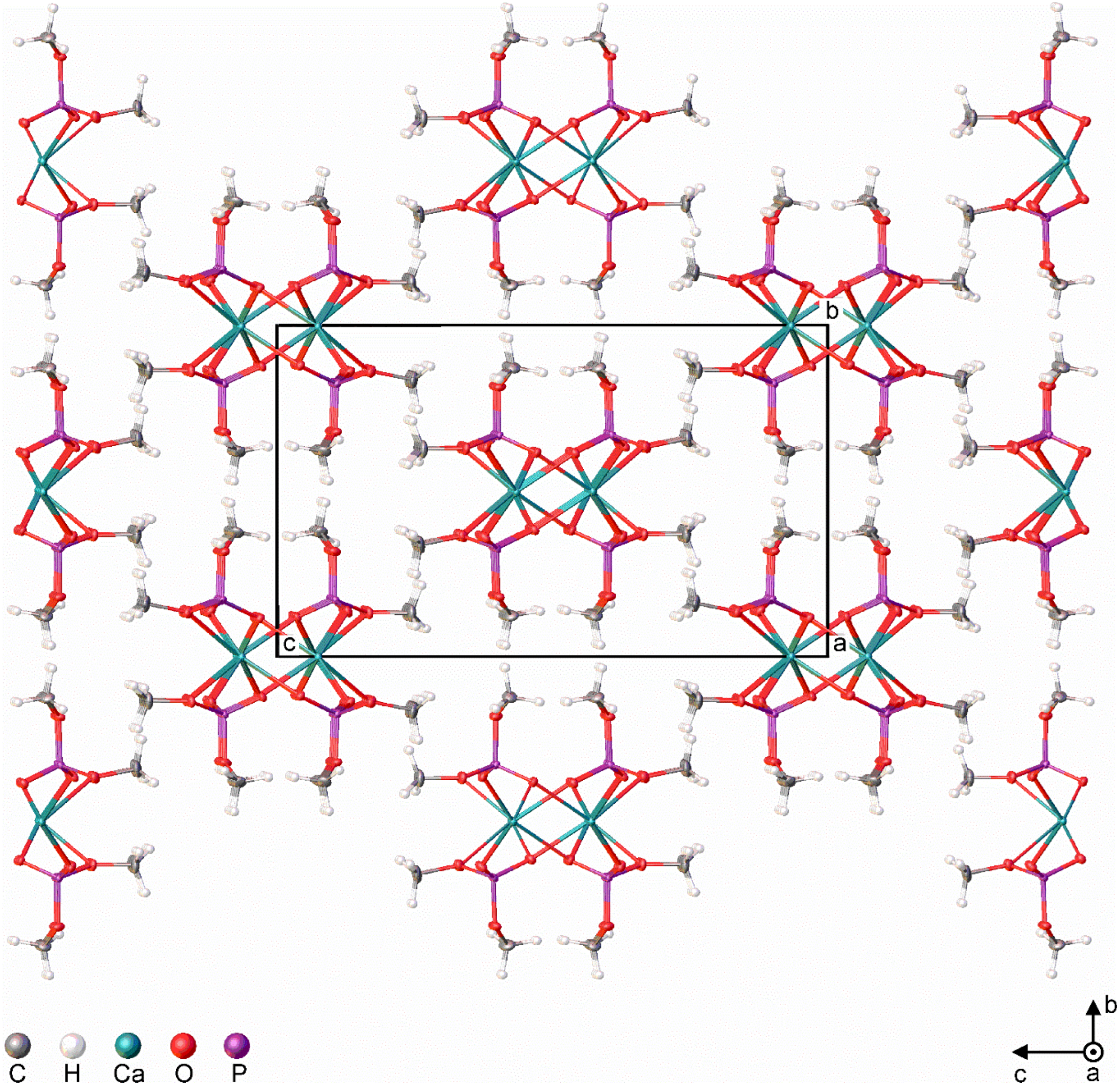

Fig. S9 Packing of CaDMP chains in a view along the crystallographic *x*-axis. The borders of CaDMP crystal unit cell are indicated with black, solid lines

*Corresponding author. maciej.debowski@pw.edu.pl

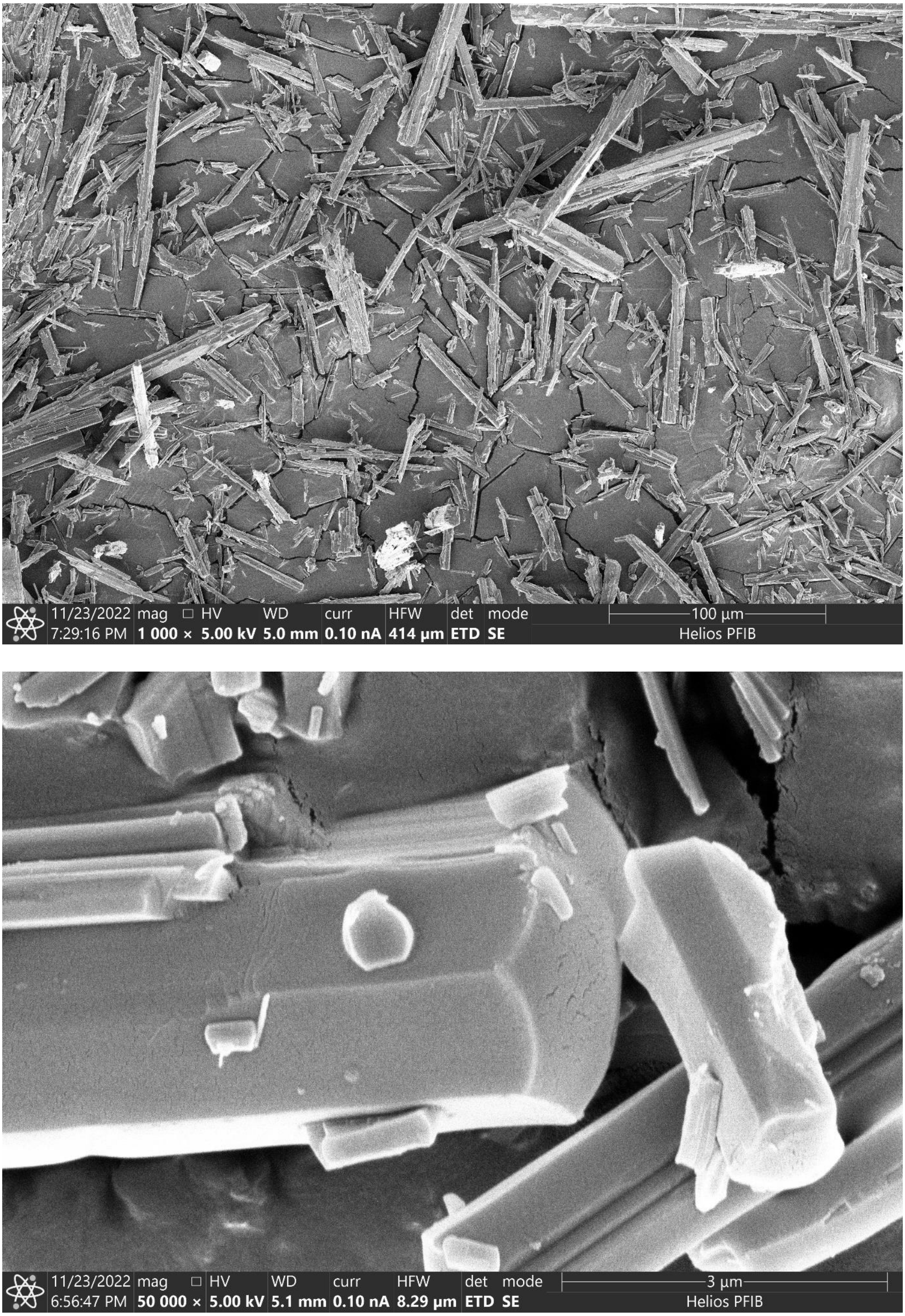

Fig. S10 SEM images of CaDMP particles. Magnification of 1,000× (*top*) and 50,000× (*bottom*)

*Corresponding author. maciej.debowski@pw.edu.pl

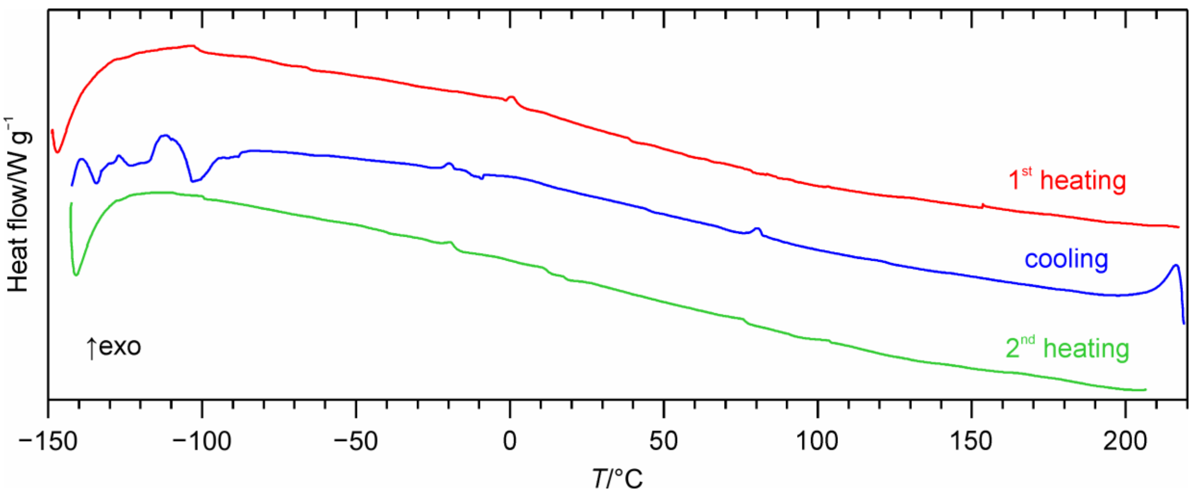

Fig. S11 DSC curves of CaDMP recorded between −150 and 220 °C (heating/cooling rates of 10 °C min^{-1})

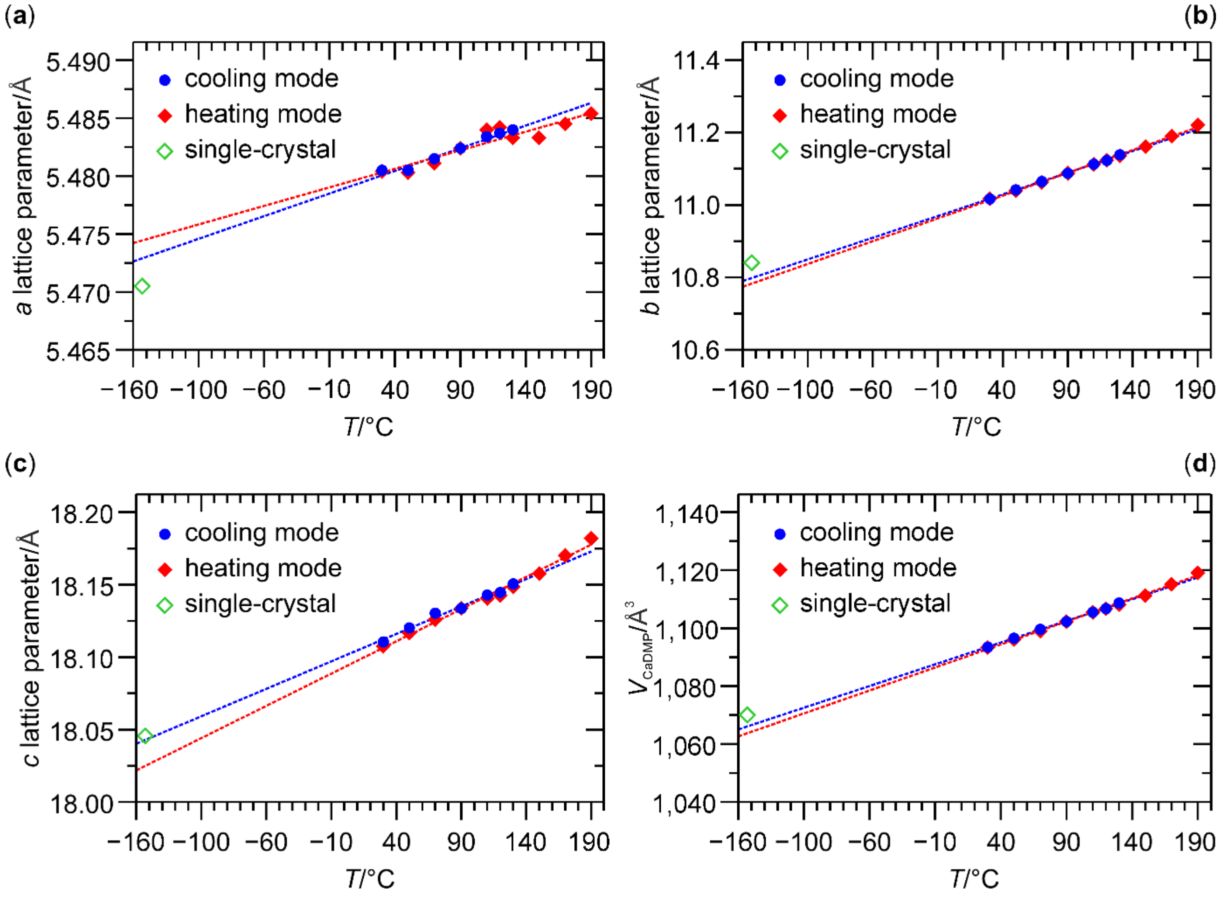

Fig. S12 The effect of temperature on the CaDMP lattice parameters *a*, *b*, *c* (**a**–**c**), and unit cell volume V_{CaDMP} (**d**). Data points derived from the VT-PXRD measurements are presented in red and blue (● and ♦ symbols for cooling and heating modes, respectively), whereas the values estimated from a single-crystal X-ray diffraction experiment are depicted as ◊ symbols. The dashed lines are drawn based on Eqs. (S6)–(S9) (for a heating mode), and Eqs. (S10)–(S13) (for a cooling mode)

*Corresponding author. maciej.debowski@pw.edu.pl

Evaluation of the relative crystallinities of the α- and β-iPP phases from DSC melting endotherms

The procedure was carried out according to a following method proposed by Li and Cheung [S6]. A bimodal melting endotherm recorded during a DSC heating cycle was integrated giving a total heat of fusion of iPP within the investigated sample ($\Delta H_{\mathrm{m}}^{\mathrm{total}}$) calculated with respect to the mass of polymer in the sample. Subsequently, a component corresponding to a melting of the β-iPP crystals ($\Delta H_{\mathrm{m},\beta}^{*}$) can be separated from the calculated $\Delta H_{\mathrm{m}}^{\mathrm{total}}$, by drawing a vertical line (h) from the maximum between the two melting peaks to the baseline, as depicted in Fig. S13.

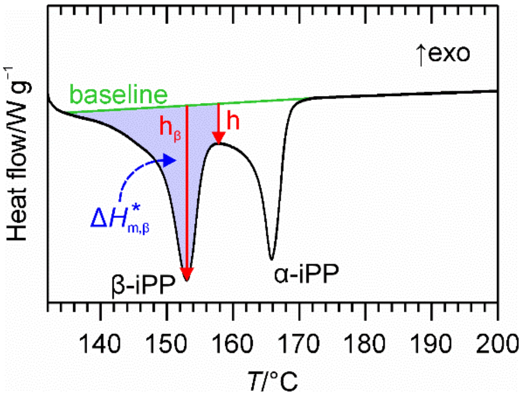

Fig. S13 DSC melting curve of the sample containing a mixture of the α- and β-iPP crystal phases. Analysis of β-iPP fraction.

A specific heat of fusion of the β-iPP phase ($\Delta H_{\mathrm{m}}^{\beta}$) could be obtained according to Eq. (S14):

$$\Delta H_{\mathrm{m}}^{\beta} = \Delta H_{\mathrm{m},\beta}^{*} \times \mathrm{f_{cal}} \tag{S14}$$

in which $\mathrm{f_{cal}}$ is a calibration factor accounting for a partial overlapping of the α- and β-iPP melting endotherm (i.e., a contribution of melting of the less perfect α-iPP crystals to $\Delta H_{\mathrm{m},\beta}^{*}$). A value of $\mathrm{f_{cal}}$ could be derived from the lengths of h and a vertical line drawn from the maximum of the β-iPP melting peak to the baseline ($\mathrm{h_\beta}$), following Eq. (S15).

$$\mathrm{f_{cal}} = \left[1 - \left(\mathrm{h}/\mathrm{h_\beta}\right)\right]^{0.6} \tag{S15}$$

A specific heat of fusion of the α-iPP phase ($\Delta H_{\mathrm{m}}^{\alpha}$) is a difference between the values of $\Delta H_{\mathrm{m}}^{\mathrm{total}}$ and $\Delta H_{\mathrm{m}}^{\beta}$. By knowing $\Delta H_{\mathrm{m}}^{\alpha}$, $\Delta H_{\mathrm{m}}^{\beta}$, and the standard heat of fusion of either the α-iPP or β-iPP crystal phase ($\Delta H_{\mathrm{m},i}^{0}$), the absolute crystallinity of each iPP polymorph within the investigated sample ($X_{\mathrm{c,m}}^{i}$) could be calculated separately according to Eq. (S16):

$$X_{\mathrm{c,m}}^{i} = \Delta H_{\mathrm{m}}^{i}/\Delta H_{\mathrm{m},i}^{0} \tag{S16}$$

where *i* stands for α or β, depending on the type of the iPP crystal phase. For these calculations values of 170 J g^{-1} ($\Delta H_{\mathrm{m},\alpha}^{0}$ [S7]) and 168.5 J g^{-1} ($\Delta H_{\mathrm{m},\beta}^{0}$ [S8]) The overall absolute degree of crystallinity of iPP within the analyzed material ($X_{\mathrm{c,m}}^{\mathrm{total}}$) could be obtained as the sum of $X_{\mathrm{c,m}}^{\alpha}$ and $X_{\mathrm{c,m}}^{\beta}$.

*Corresponding author. maciej.debowski@pw.edu.pl

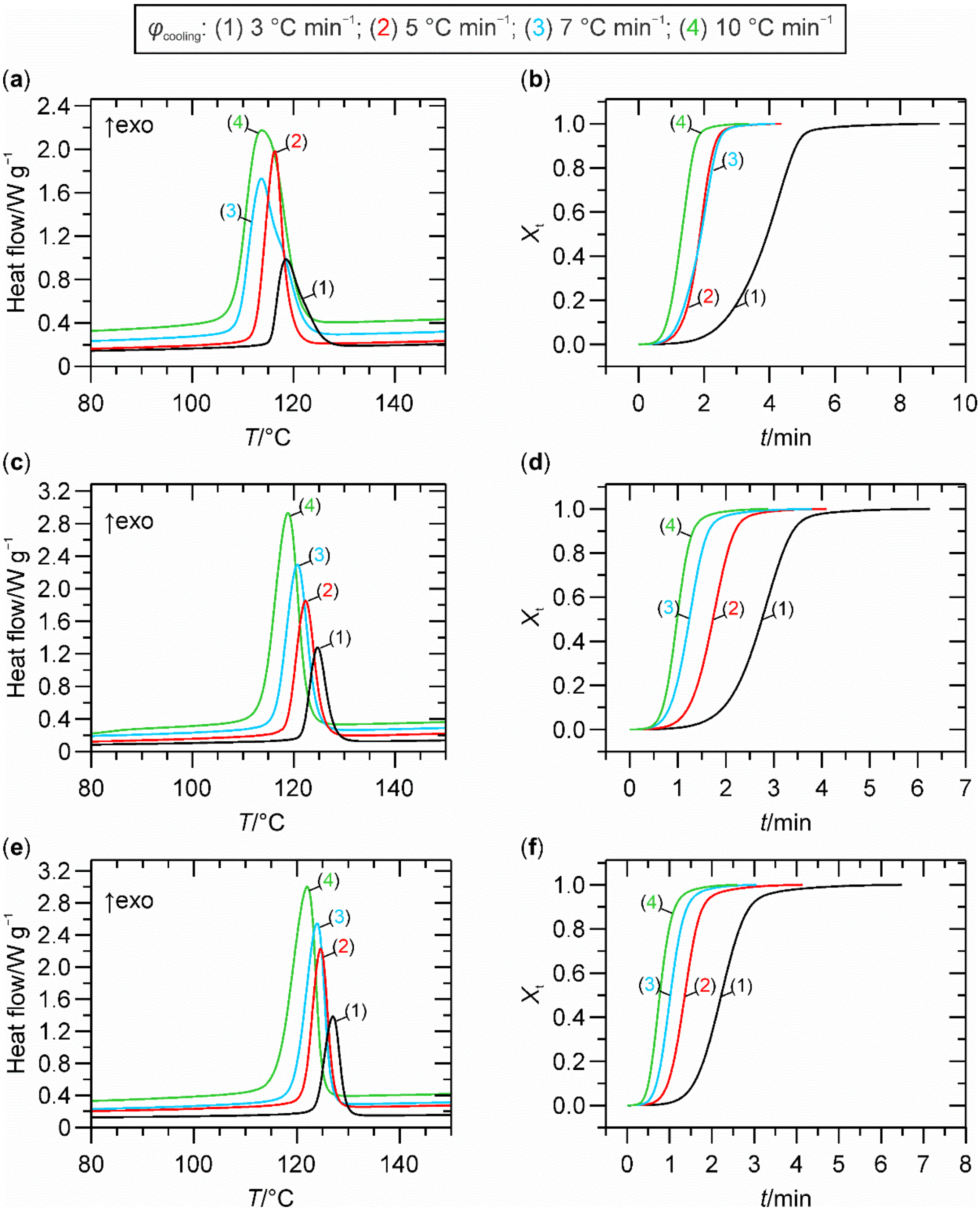

Figure S14. Non-isothermal crystallization of neat iPP (**a**, **b**) and its composites with 0.2 wt% (**c**, **d**) or 1.0 wt% (**e**, **f**) of CaDMP. DSC curves recorded during cooling with different cooling rates (*left side*) and the variation of a relative crystallinity with crystallization time (*right side*). Applied cooling rates: (1) 3 °C min^{-1}; (2) 5 °C min^{-1}; (3) 7 °C min^{-1}; (4) 10 °C min^{-1}

*Corresponding author. maciej.debowski@pw.edu.pl

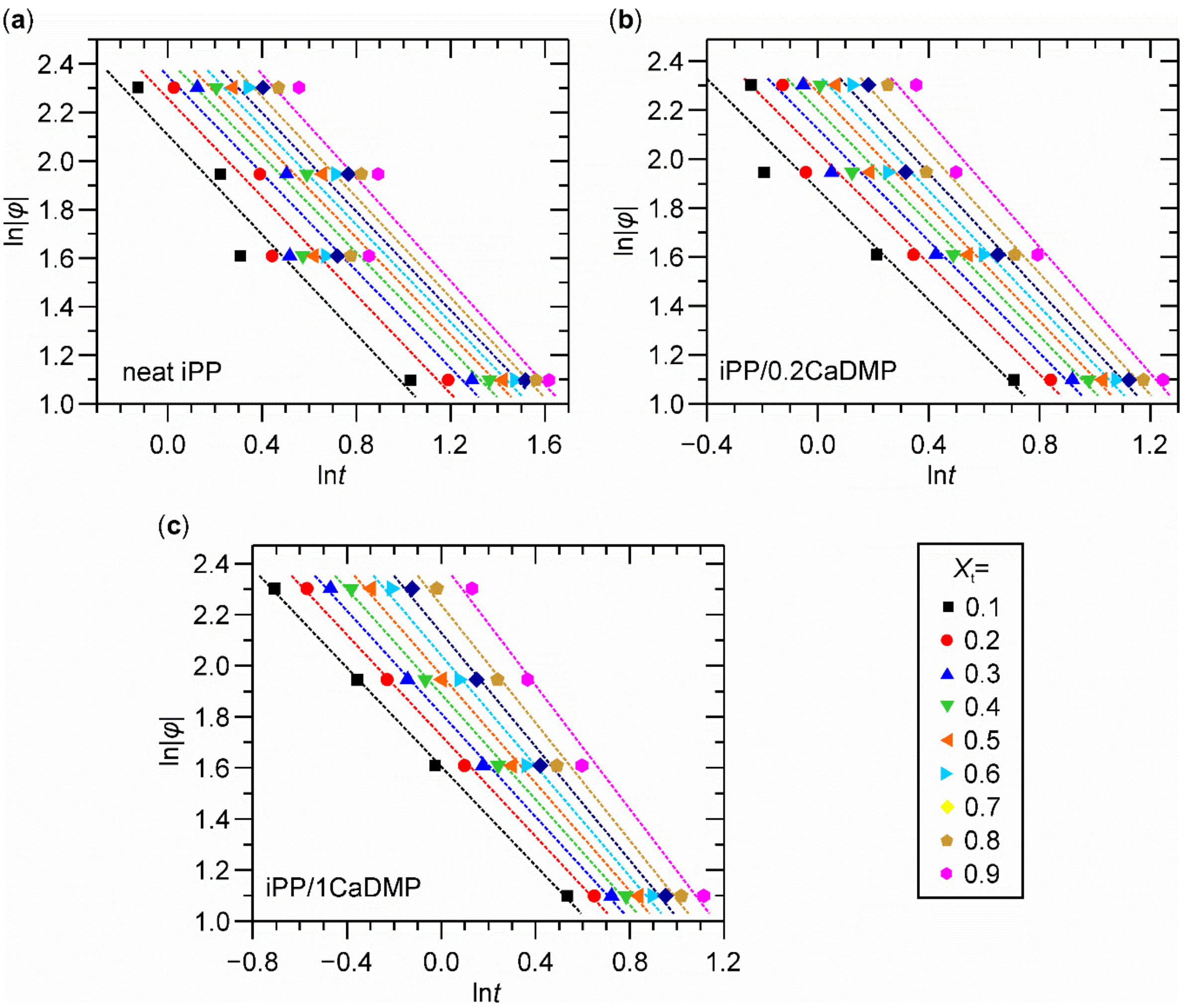

Fig. S15 The Liu and Mo plots for: (**a**) iPP and its composites filled with (**b**) 0.2 wt% or (**c**) 1.0 wt% of CaDMP

*Corresponding author. maciej.debowski@pw.edu.pl

Elaboration of the experimental WAXS data

WAXS curves, corrected for background scattering and normalized to the same integrated intensity I in the whole 2θ angle range, were deconvoluted into separate components arising from the crystalline and amorphous phases, using a WAXFIT computer program [S9, S10]. The deconvolution was performed by means of an approximation method, which included a construction of a theoretical curve composed of the functions related to the individual reflections of a crystalline phase (or phases) and amorphous maxima. The component functions were linear combinations of the Gauss and Cauchy functions. The parameters of these functions were found by the best fitting of the resulting theoretical curve to the experimental one, using a suitable optimization procedure[S9, S11]. Fig. S16 shows the results of a deconvolution procedure applied to the WAXS profiles recorded for neat iPP and its composite with 1 wt% of CaDMP.

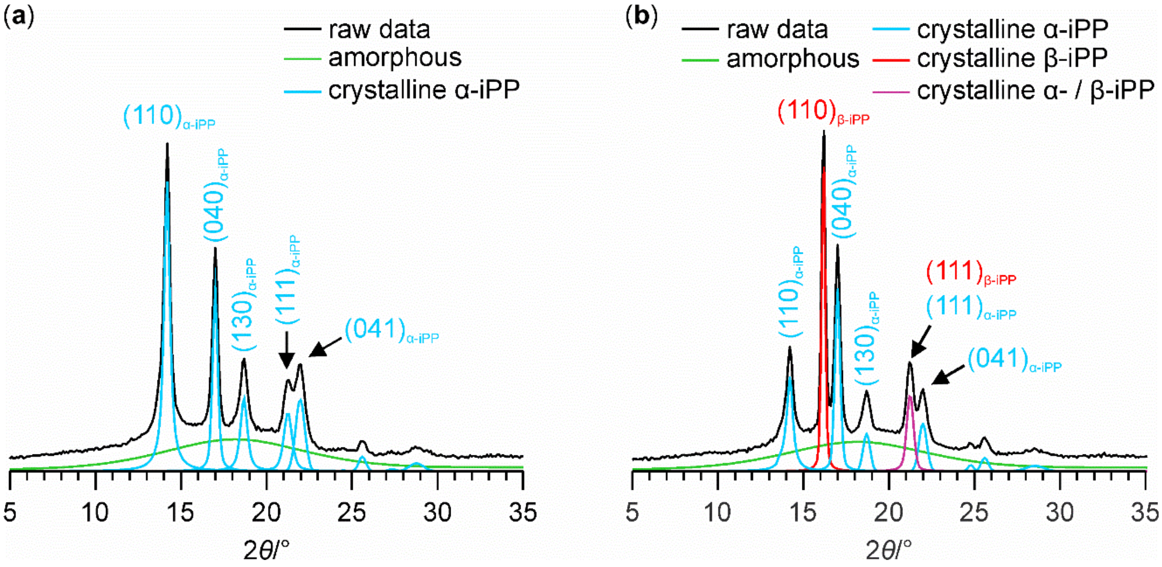

Fig. S16 X-ray diffraction reflections of isotactic polypropylene from a deconvolution of the experimental (raw) WAXS curves recorded for: (**a**) neat iPP, (**b**) composite of iPP with 1 wt% of CaDMP. Miller indices of the crystallographic planes ascribed to the reflections with the highest intensities are denoted according to the literature data [S12]

A total degree of crystallinity of each sample (X_c^{WAXS}) was calculated as a ratio of the integral intensity of its all crystalline reflections to the total integral intensity of the X-ray radiation scattered by it in the whole 2θ angle range, following Eq. (S17):

$$X_c^{WAXS} = \sum I_c^{\mathrm{WAXS}} / (\sum I_c^{\mathrm{WAXS}} + \sum I_a^{\mathrm{WAXS}}) \quad \text{(S17)}$$

where I_c^{WAXS} and I_a^{WAXS} are the integral intensities of the crystalline reflections and the amorphous maxima, respectively.

*Corresponding author. maciej.debowski@pw.edu.pl

SUPPORTING INFORMATION REFERENCES

[S1] Brown ID, Altermatt D. Bond-valence parameters obtained from a systematic analysis of the Inorganic Crystal Structure Database. Acta Crystallogr Sect B: Struct Sci. 1985;41:244–7. https://doi.org/10.1107/S0108768185002063

[S2] Brese NE, O'Keeffe M. Bond-valence parameters for solids. Acta Crystallogr Sect B: Struct Sci. 1991;47:192–7. https://doi.org/10.1107/S0108768190011041

[S3] Brown ID. Bond Valence Parameters. In: Crystallographic data compilations. International Union of Crystallography. https://www.iucr.org/__data/assets/file/0011/150779/bvparm2020.cif. Accessed 9 Feb 2024.

[S4] Zachara J. Novel Approach to the Concept of Bond-Valence Vectors. Inorg Chem. 2007;46:9760–7. https://doi.org/10.1021/ic7011809.

[S5] Dębowski M, Florjańczyk Z, Ostrowski A, Guńka PA, Zachara J, Krztoń-Maziopa A, Chazarkiewicz J, Iuliano A, Plichta A. 1D and 2D hybrid polymers based on zinc phenylphosphates: synthesis, characterization and applications in electroactive materials. RSC Adv. 2021;11:7873–85. https://doi.org/10.1039/D0RA09493E

[S6] Li JX, Cheung WL. On the deformation mechanisms of β-polypropylene: 1. Effect of necking on β-phase PP crystals. Polymer 1998;39:6935–40. https://doi.org/10.1016/S0032-3861(98)00144-X

[S7] Lanyi FJ, Wenzke N, Kaschta J, Schubert DW. On the Determination of the Enthalpy of Fusion of α-Crystalline Isotactic Polypropylene Using Differential Scanning Calorimetry, X-Ray Diffraction, and Fourier-Transform Infrared Spectroscopy: An Old Story Revisited. Adv Eng Mater. 2020;22:1900796. https://doi.org/10.1002/adem.201900796

[S8] Li JX, Cheung WL, Jia D. A study on the heat of fusion of β-polypropylene. Polymer 1999;40:1219–22. https://doi.org/10.1016/S0032-3861(98)00345-0

[S9] Rabiej M, Rabiej S. Analysis of Synchrotron WAXD Curves of Semicrystalline Polymers by Means of the Optifit Computer Program. Fibres & Textiles in Eastern Europe 2005;13:75–8.

[S10] Rabiej M. Application of a multicriterial optimization to the resolution of X-ray diffraction curves of semicrystalline polymers. Polimery 2017;62:821–6. https://doi.org/10.14314/polimery.2017.821

[S11] Rabiej M. Application of the particle swarm optimization method for the analysis of wide-angle X-ray diffraction curves of semicrystalline polymers. J Appl Cryst. 2017;50:221–30. https://doi.org/10.1107/S160057671601983X

[S12] Auriemma F, De Rosa C, Malafronte A, Scoti M, Di Girolamo R. Solid State Polymorphism of Isotactic and Syndiotactic Polypropylene. In: Karger-Kocsis J, Bárány T, editors. Polypropylene Handbook. Cham: Springer Nature Switzerland AG; 2019. pp. 37–119.

*Corresponding author. maciej.debowski@pw.edu.pl
